# Identification of m^6^A Regulator-Associated Methylation Modification Clusters and Immune Profiles in Melanoma

**DOI:** 10.3389/fcell.2021.761134

**Published:** 2021-12-21

**Authors:** Fengying Du, Han Li, Yan Li, Yang Liu, Xinyu Li, Ningning Dang, Qingqing Chu, Jianjun Yan, Zhen Fang, Hao Wu, Zihao Zhang, Xingyu Zhu, Xiaokang Li

**Affiliations:** ^1^ Department of Dermatology, Central Hospital Affiliated to Shandong First Medical University, Jinan, China; ^2^ Department of Gastroenterological Surgery, Shandong Provincial Hospital, Cheeloo College of Medicine, Shandong University, Jinan, China; ^3^ Medical Science and Technology Innovation Center, Shandong First Medical University and Shandong Academy of Medical Sciences, Jinan, China; ^4^ Department of Gastroenterological Surgery, The First Affiliated Hospital of Shandong First Medical University, Jinan, China; ^5^ Department of Respiratory and Critical care, Shandong public health clinical center, Jinan, China; ^6^ Department of Oncology, Shandong Provincial Hospital Affiliated to Shandong First Medical University, Jinan, China; ^7^ Department of Dermatology, Shandong Provincial Hospital Affiliated to Shandong First Medical University, Jinan, China; ^8^ Outpatient of Podiatric Rehabilitation, Maternity and Child Health Care of Zaozhuang, Zaozhuang, China; ^9^ Department of Dermatology, Qilu Hospital, Shandong University, Jinan, China; ^10^ Department of General Surgery, Xuanwu Hospital, Capital Medical University, Beijing, China

**Keywords:** skin cutaneous melanoma, methylation of N6 adenosine modification, tumor microenvironment, immune profiles, immunotherapy

## Abstract

RNA N6-methyladenosine (m^6^A) modification in tumorigenesis and progression has been highlighted and discovered in recent years. However, the molecular and clinical implications of m^6^A modification in melanoma tumor microenvironment (TME) and immune infiltration remain largely unknown. Here, we utilized consensus molecular clustering with nonnegative matrix factorization based on the melanoma transcriptomic profiles of 23 m^6^A regulators to determine the m^6^A modification clusters and m^6^A-related gene signature. Three distinct m^6^A modification patterns (m^6^A-C1, C2, and C3), which are characterized by specific m^6^A regulator expression, survival outcomes, and biological pathways, were identified in more than 1,000 melanoma samples. The immune profile analyses showed that these three m^6^A modification subtypes were highly consistent with the three known immune phenotypes: immune-desert (C1), immune-excluded (C2), and immune-inflamed (C3). Tumor digital cytometry (CIBERSORT, ssGSEA) algorithm revealed an upregulated infiltration of CD8^+^ T cell and NK cell in m^6^A-C3 subtype. An m^6^A scoring scheme calculated by principal component of m^6^A signatures stratified melanoma patients into high- and low-m^6^sig score subgroups; a high score was significantly associated with prolonged survival and enhanced immune infiltration. Furthermore, fewer somatic copy number alternations (SCNA) and PD-L1 expression were found in patients with high m^6^Sig score. In addition, patients with high m^6^Sig score demonstrated marked immune responses and durable clinical benefits in two independent immunotherapy cohorts. Overall, this study indicated that m^6^A modification is involved in melanoma tumor microenvironment immune regulation and contributes to formation of tumor immunogenicity. Comprehensive evaluation of the m^6^A modification pattern of individual tumors will provide more insights into molecular mechanisms of TME characterization and promote more effective personalized biotherapy strategies.

## Introduction

Methylation of N6 adenosine (m^6^A) is a revisable RNA modification process that is widely present in various types of common RNAs, such as mRNAs, lncRNAs, and miRNAs, and essential for a variety of physiological processes and disease progression (Zhao et al., 2017; Zaccara et al., 2019). The m^6^A modification is manipulated by three regulatory proteins, methyltransferases (“writers”), demethylases (“erasers”), and binding proteins (“reader”), and this modification process is dynamic and reversible (He et al., 2019). Increasing evidence has identified the important roles m6A modifications play in various cellular processes and in cancer progression through regulating RNA stability, mRNA splicing and translation, and microRNA processing (Li et al., 2019; Chen et al., 2020a). Meanwhile, a large number of studies have shown that the process of tumor development and abnormal immune regulation of the body are associated with abnormal expression of m^6^A-modified regulatory proteins (Chen et al., 2019a; Shulman and Stern-Ginossar, 2020; Wang et al., 2020). Therefore, systematic and comprehensive explanation of tumor heterogeneity brought about by genetic variation and epigenetic regulation will facilitate the development and advancement of new therapeutic technologies based on RNA methylation (Martínez-Riaño et al., 2019).

Malignant melanoma is a highly metastatic cancer caused by abnormal transformation of pigment cells and melanocytes resulting from prolonged exposure to ultraviolet radiation (Mazurkiewicz et al., 2021). Since melanoma is curable in its initial stages, early diagnosis of this disease is crucial (Eddy and Chen, 2020). Global data show that patients with melanoma who develop metastases have a 5-years survival rate of only 25% due to the difficulty of treatment (Eddy and Chen, 2020). Multiple novel targeted therapies targeting melanoma-specific markers have been developed in recent years; however, most patients often show lower effectiveness or shorter duration to these treatments (Mazurkiewicz et al., 2021). Among the multiple factors that influence treatment outcome, the tumor microenvironment might account for a major cause in the melanoma progression. The composition of the microenvironment in melanoma is relatively complex, which includes adventitial cells (keratin-forming cells, cancer-associated fibroblasts CAF, adipocytes and infiltrating immune cells), extracellular matrix components, and tumor-specific physicochemical properties (Mazurkiewicz et al., 2021). With the increased understanding of the tumor microenvironment, the key immune cell subsets in tumorigenesis and metastasis were also gradually recognized. The evaluation of immune infiltration based on the characteristics of TME was supposed as a key technique to infer the pre-existing antitumor immunity and predict patient response to immune checkpoint inhibitor therapy (Binnewies et al., 2018; Galon and Bruni, 2019; Li et al., 2020a). Recently, the new concept of “immune context” on tumor, which classifies the TME characteristics of melanoma into three categories, i.e., hot, excluded, and cold, also implies three different types of effective treatment options (Hegde et al., 2016; Chen and Mellman, 2017). In summary, systematic and comprehensive dissection of the components of the tumor microenvironment of melanoma and thus identification of the corresponding tumor immune phenotype is a feasible and reliable means to guide immunotherapy and predict the effectiveness of immunotherapy (Mariathasan et al., 2018; Pagès et al., 2018).

Recent studies suggest an association between TME immune cell infiltration and m^6^A modification; however, this does not appear to be fully explained by RNA degradation mechanisms (Zhao et al., 2017; Chen et al., 2019a; He et al., 2019). It has been reported that YTHDF1 can promote lysozyme in dendritic cells to regulate the degradation of tumor neoantigens, and the key to this process is that YTHDF1 can accurately recognize the m^6^A modification process of tumor neoantigens and enhance their translation level (Han et al., 2019). When YTHDF1 is absent in dendritic cells, this leads to enhanced cross-presentation of antigens and enhanced cross-stimulation of CD8^+^ T cells. FTO has been reported to be associated with cytotoxic effects in colon cells by inhibiting YTHDF2-mediated RNA decay, which in turn promotes PD-1, CXCR4, and SOX10, and suppresses interferon-gamma (IFN-γ) expression (Yang et al., 2019). This result was confirmed in an *in vitro* experiment. When FTO is knocked down exogenously, IFN-γ is substantially upregulated, which in turn makes colon cancer mice sensitive to anti-PD-1 drug treatment. METTL3, which also regulates mRNA m^6^A modifications, regulates the dynamic balance of CD40, CD80, and Snail (Lewinska et al., 2017; Wang et al., 2019). Unfortunately, due to the unsophisticated nature of the current technology, the studies mentioned are all on one or two m^6^A regulatory molecules, and the antitumor effects produced by these regulatory molecules are not the contribution of one or several molecules, but rather they work together to regulate the m^6^A modification process in the body and thus affect cancer development and metastasis. Fortunately, the explosive growth on transcriptomics and genomics sequencing database provides a rich resource for a comprehensive and integrated analysis of the role of m^6^A-related molecules in cancer and immune regulation (Finotello and Trajanoski, 2018; Chen et al., 2020b). Thus, deepening our understanding of cancer immunity and developing new targets for cancer immunotherapy requires a systematic and comprehensive dissection of the TME immune cell infiltration profile regulated by m^6^A-related molecules.

In this study, we integrated the transcriptome and genome sequencing data from 1,020 melanoma samples across TCGA and GEO databases, and systematically analyzed and discovered the direct and specific association between m^6^A modification patterns and TME immune cell infiltration features in melanoma. Using non-negative matrix factorization (NMF) clustering analysis, we identified three novel m^6^A modification patterns with TME features highly consistent with three previously reported immune phenotypes: immune inflammatory, immune rejection, and immune desert phenotypes (Chen and Mellman, 2017). Not only that, we quantified the m^6^A modification clusters of individualized tumors in the form of scores, which can be used to predict the effectiveness of patients to ICI therapy. Our findings suggest that m^6^A modifications play a crucial role in tumor immune microenvironment signature formation and melanoma treatment planning.

## Materials and Methods

### Collect and Preprocess of Publicly Attainable Expression Datasets

Gene expression data and clinical information for melanoma patient samples were obtained from the GEO database (https://www.ncbi.nlm.nih.gov/geo/) and TCGA database (https://portal.gdc.cancer.gov/), which are publicly and freely available. We screened the melanoma dataset and eventually included a total of 1,020 patients in the study for subsequent analysis, including the GSE19234, GSE22154, GSE50509, GSE59455, GSE65904, GSE22153, GSE54437, and TCGA-SCKM datasets. For data pre-processing, we downloaded the “CEL” files from the GEO database, relying on the “affy” and “simpleaffy” R packages for background correction and normalization, while the RNA sequencing data from the TCGA database is downloaded in normalized FPKM format and then converted to transcripts per kilobase million (TPM) format. With reference to previous experience, the “ComBat” method of the “sva” R package was used to reduce the batch effect between different datasets, which was mainly a problem for datasets from the GEO database (Dai et al., 2018). Both somatic mutation data and copy number variation data of TCGA-SCKM were curated from the UCSC Xena database and Davoli et al. (Davoli et al., 2017). The copy number variation of 23 m^6^A regulators in human chromosomes was mapped by the “RCircos” R package. For non-synonymous mutations such as frameshift mutations, inflammatory mutations, missense mutations, nonsense mutations, and splice site mutations, numbers represent the tumor mutational load (TML). Supplementary Table S1 presents the clinical information of the samples from the *meta*-GEO and TCGA-SKCM databases.

### Nonnegative Matrix Factorization Clustering Analysis of 23 m^6^A Regulators

A literature review of m^6^A methylation modifications revealed that there are now 23 recognized m^6^A regulators, which constitute the modification pattern of m^6^A methylation (Zhao et al., 2017; Chen et al., 2019a; He et al., 2019; Zaccara et al., 2019). Specifically, eight writers include CBLL1, KIAA1429, METTL14, METTL3, RBM15, RBM15B, WTAP, and ZC3H13; two erasers include ALKBH5 and FTO; 13 readers include ELAVL1, FMR1, HNRNPA2B1, HNRNPC, IGF2BP1, IGF2BP2, IGF2BP3, LRPPRC, YTHDC1, YTHDC2, YTHDF1, YTHDF2, and YTHDF3; and 23 of them shared the key task of m^6^A methylation modification. Using non-negative matrix decomposition (NMF), we performed a clustering analysis of the 23 m^6^A regulators based on their expression, which could identify different types of m^6^A modification patterns. The expression matrix A of the 23 m^6^A regulators was first split into non-negative matrices W and H, as A ≈ WH, and then the matrix A was subjected to repeated factorization, and finally the output data was summarized, which gave the clustering results of the melanoma samples. It is crucial to consider factors such as covariance, dispersion, and silhouette coefficient to determine the optimal number of clustering groups. The “NMF” R package to perform the clustering analysis used the “brunet” and “200 nruns” algorithms.

### Functional Analysis and Annotation

The Hallmarker gene set (Subramanian et al., 2005) and Mariathasan et al. (Mariathasan et al., 2018) constructed gene set were used as well-defined biometric backgrounds for gene set variation analysis (GSVA) with “GSVA” R package (Hänzelmann et al., 2013), which was designed to explore the variation in biological processes across different m^6^A modification patterns. In the gene ontology (GO) analysis, we annotated the functions of 23 m^6^A regulators under three entries of cellular component (CC), molecular function (MF), and biological process (BP), which was done using the “clusterProfiler” R package. For GSVA and GO analysis, the cut-off value was set to a false discovery rate (FDR) < 0.01.

### Estimation of Immune Cell Infiltration

In quantifying the relative abundance of 28 immune cell types curated by Charoentong et al. in the tumor microenvironment, we refer to recent studies using the single sample gene enrichment analysis (ssGSEA) method, which well marks the specific functional gene panels of each immune cell type (Charoentong et al., 2017; Jia et al., 2018). As in the previous study (Chen et al., 2020a), we expressed the relative abundance of various immune cell types in the form of enrichment scores, and they were normalized to a uniform distribution from 0 to 1. In terms of biosimilarity, infiltrating immune cells were evaluated and acted upon using multidimensional scaling (MDS) and Gaussian fitting models, and moreover, the deconvolution approach CIBERSORT (Newman et al., 2019) (http://cibersort.stanford.edu/) was then used to estimate the abundance of 22 different subpopulations of leukocytes, which have melanoma gene expression profiles.

### Quantification of Immune Response Predictor

T cell-inflamed gene expression profile (GEP) is a superior predictor of response to anti-PD-1 regimens, which contained IFN-γ-responsive genes related to antigen presentation, cytotoxic activity, and adaptive immune resistance (Ayers et al., 2017). The T cell-inflamed scores were calculated and weighted by averaging of the included genes for the IFN-γ (6-gene) and expanded immune (18-gene) signatures. In modeling different types of tumor immune evasion mechanisms, we drew on the Tumor Immune Dysfunction and Exclusion (TIDE) algorithm proposed by Jiang et al. (Jiang et al., 2018). This algorithm integrates the dysfunction of tumor-infiltrating toxic T lymphocytes (CTLs) and rejection of CTLs by immunosuppressive factors. The higher TIDE score implies greater chance of immune escape of tumor cells and represents a possible poor outcome of treatment with ICIs. The method of Estimation of Stromal and Immune cells in MAlignant Tumor tissues using Expression data (ESTIMATE) (Yoshihara et al., 2013) was adopted to calculate the immune score of tumors, and this algorithm can be better based on transcriptional profiles to estimate the cellularity of the tumor and the purity of the tumor. The level of infiltrating immune and stromal cells is the basis for tumor purity, which is predicted by the immune score of the tumor. In detail, a high immune score of a tumor is an indication of a high infiltration of immune cells in the tumor tissue, or a low tumor purity.

### Capture of Significantly Mutated Genes and Tumor Mutation Features

The MutSigCV algorithm was used to identify significantly mutated genes (SMGs) (Lawrence et al., 2013; Chen et al., 2019b), which takes into account the specific background mutation rate in the mutation context before evaluating the significant enrichment of non-resting somatic mutations in a gene. We considered q < 0.1 as statistically significant, and these genes needed to be certified in the Cancer Cell Line Encyclopedia of Humans (CCLE) (Ghandi et al., 2019) to be defined as SMGs (Chen et al., 2020c) (Supplementary Table S2). The “maftools” R package (Mayakonda et al., 2018) was used to characterize genes in the TCGA-SKCM cohort that underwent m^6^A modification, the mutation details of SMGs, and the capture of mutational features in the genomic data. The ExtractSignatures function based on Bayesian variation non-negative matrix decomposition was used for model construction; specifically, using this function, we split the mutation portrait matrix into two non-negative matrices and noted as “signature” and “contribution,” where “signature” represents the mutation process and “contribution” represents the corresponding mutation activities (Chong et al., 2021a). Better still, the SignatureEnrichment function allows determining the optimal number of extracted mutation features and assigning them appropriately to each sample. For comparison and annotation, using the Catalogue of Somatic Mutations in Cancer (COSMIC) (Kandoth et al., 2013) as a reference, we performed a cosine similarity analysis on the extracted melanoma mutation portraits.

### Identify Differentially Expressed Genes Between Different m^6^A Modification Phenotypes

Patients were classified into three clusters of m^6^A modification patterns using a consensus clustering algorithm, and then the “limma” R package (Ritchie et al., 2015) was used to find differentially expressed genes between groups. Voom normalized data were then subjected to “lmFit” and “eBayes” function algorithms, which in turn allowed the calculation of specific data for differential expression. In this process, we set adjusted *p*-values <0.001 as statistically significant differences.

### Construct the m^6^Sig Score System

Based on principal component analysis (PCA), we constructed an m^6^A score system to quantify the level of m^6^A modifications in specific patients. According to DEGs, they are the intersecting parts of different m^6^A clusters, and we analyzed the prognostic impact of each gene on melanoma patients with the help of univariate Cox regression models. Deeper feature selection was performed for genes that significantly affect the prognosis of melanoma patients, and this process was computed by the recursive feature elimination (RFE) method of random forest and the 10-fold cross-validation method included in the “caret” R package. Further, we obtained the gene expression profiles based on the above steps, and the principal components 1 and 2 obtained from PCA analysis were the basis of our feature score. The specific formula for this score system is referred to a previous study (Zhang et al., 2020; Chong et al., 2021b), m^6^Sig score = ∑(PC1i + PC2i).

### Collect Genomic and Clinical Information for the ICI Cohort

The gene expression profiles of patients treated with ICI were retrieved in publicly available databases, focusing on matching with clinical information. Ultimately, we included metastatic melanoma treated with PD-1 (nivolumab or pembrolizumab) or PD-1 combined with CTLA-4 (ipilimumab) (Liu et al., 2019), and metastatic urothelial carcinoma (mUC) treated with atezolizumab (anti-PD-L1 mcAb) (Mariathasan et al., 2018) in this study. The gene expression profiles of the samples were converted in TPM format.

### Statistical Analyses

All statistical analyses in the study were performed with R 3.6.1. Student’s t-test was performed for quantitative data conforming to a normal distribution, and Wilcoxon rank sum test was performed for non-normally distributed data. When more than two sets of analyses were performed, the nonparametric test was the Kruskal-Wallis test, while the parametric test was the analysis of variance (Hazra and Gogtay, 2016). The Fisher exact test was used for the calculation of contingency rates. Kaplan-Meier survival analysis and Cox regression analysis were performed using the “Survminer” package, and the m^6^Sig score subgroup stratum was “survival” package with the surv-cutpoint function completed. “timeROC” package completed the evaluation of the m^6^Sig score model, which plotted the corresponding subject operating characteristic curve (ROC) and calculated the area under the curve (AUC). In analyzing the relationship between patient’s clinical characteristics and the m^6^Sig score system, multivariate regression models were used to adjust for confounding factors in this. *p* < 0.05 was considered as statistical significance, and the Benjamini-Hochberg method was used to perform multiple hypothesis testing for false discovery rate (FDR) (Love et al., 2014).

## Results

### Mapping Genetic Variants of m^6^A Regulators in Melanoma

**FIGURE 1:**
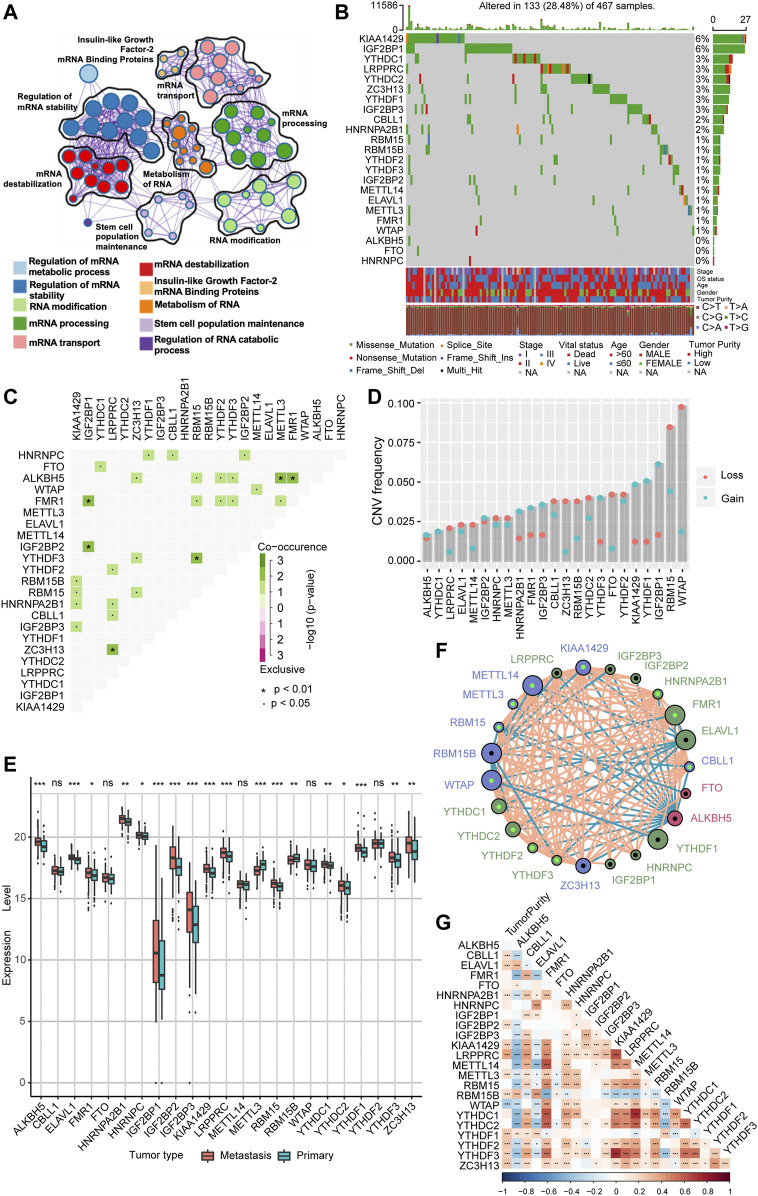
The landscape of genetic alterations of m^6^A regulators in melanoma. **(A)** Visualization of the Metascape enrichment network presenting similarities within and between clusters of terms. The same colors represent the same clustering terms. **(B)** Mutations in 23 m^6^A regulators were present in 133 of 467 melanoma patients (28.48%), with the most prevalent missense mutations, nonsense mutations, and frame shift deletion mutations. The numbers on the right side are representative of the mutation frequency of each regulator. Each column is one patient. **(C)** Visualization of co-occurrence and exclusion of 23 m^6^A regulator mutations. Green color represents co-occurrence, and purple color represents exclusion. **(D)** CNV mutations are present in all 23 m^6^A regulators. Column heights represent mutation frequencies. Pink dots represent loss mutations, and blue dots represent gain mutations. **(E)** Differential expression of mRNA of 23 m^6^A regulators in metastatic melanoma and primary melanoma. * represents *p*-values in statistics (*p < 0.05; **p < 0.01; ***p < 0.001). **(F)** Interaction network of the three m^6^A regulators in melanoma. Different colors represent different types of m^6^A regulators; green is a reader, blue is a writer, and red is an eraser. The connecting lines represent the correlation matrix; pink is positive correlation, while blue is negative correlation. Larger circles represent smaller *p*-values for prognostic analysis, and the shiny green dot in the center of the circle represents protective factors, while the black dot represents risk factors. **(G)** Visualization of tumor purity and 23 m^6^A regulator. Red color represents co-occurrence, and blue color represents exclusion.

In our study, we explored the possible physiological roles of 23 m^6^A methylation-regulated genes in melanoma, including the “writers” CBLL1, KIAA1429, METTL14, METTL3, RBM15, RBM15B, WTAP, and ZC3H13; the “readers” ELAVL1, FMR1, HNRNPA2B1, HNRNPC, IGF2BP1, IGF2BP2, IGF2BP3, LRPPRC, YTHDC1, YTHDC2, YTHDF1, YTHDF2, and YTHDF3; and the “erasers” ALKBH5 and FTO. These m^6^A regulators not only recognize, remove, and add m^6^A modification sites but also, as revealed by GO enrichment analysis and Metascape analysis, can actually alter biological processes, such as regulating mRNA stability, RNA modifications, and RNA metabolism (Figure 1A). Among 467 melanoma patient samples with genomic sequencing, 133 (28.48%) had somatic mutations in m^6^A regulators, which mainly included missense mutations, nonsense mutations, and code-shifting mutations (Figure 1B). KIAA1429 had the highest mutation frequency, followed closely by IGF2BP1, and the next in the gradient were YTHDC1, LRPPRC, YTHDC2, ZC3H13, YTHDF1, and IGF2BP3. Interestingly, IGF2BP1, ZC3H13, and YTHDF1 had only missense mutations in the relatively high mutation frequencies. Analysis of the co-mutation profiles of the 23 m^6^A regulators revealed significant co-mutations between FMR1 and IGF2BP1, IGF2BP2 and IGF2BP1, ZC3H13 and LRPPRC, YTHDF3 and RBM15, ALKBH5 and METTL3, and ALKBH5 and FMR1 (Figure 1C). When performing CNV mutation analysis, we concluded that there was widespread CNV amplification in IGF2BP1, YTHDF1, and KIAA1429, while CNV deletion was more widespread in WTAP and RBM15 (Figure 1D). Comparing primary melanoma and metastases, we found that ALKBH5, ELAVL1, FMR1, HNRNPA2B1, HNRNPC, IGF2BP1/2/3, KIAA1429, LRPPRC, RBM15, YTHDC1/2, YTHDF1/3, and ZC3H13 were significantly upregulated in metastases, while RBM15B and METTL3 were significantly upregulated in primary melanoma (Figure 1E). The expression of m^6^A regulators with aberrant CNV amplification was also upregulated in metastases compared to primary melanoma (ALKBH5, FMR1, HNRNPA2B1, IGF2BP1/2/3, KIAA1429, YTHDC1, YTHDF1/3), and conversely, m^6^A regulators with aberrant CNV deletion were also downregulated (METTL3, RBM15B, YTHDC2), which are obtained by combining Figure 1D. The m^6^A regulator network mapped in Figure 1F showed the interaction relationships between 23 molecules that are interconnected and influence each other, which further modulates the prognosis of melanoma patients. This implies that there is a complex and well-organized crossover network between the regulators of writers, readers, and erasers, and this network allows the m^6^A modification pattern to further refine and take effect, influencing the development and metastasis of melanoma. Using the Spearman correlation test, we found that there is a mutual regulatory relationship between these m^6^A regulators. Interestingly, ALKBH5 was negatively correlated with most of the m^6^A regulators, while FMR1 and HNRNPA2B1 were positively correlated with most of them (Supplementary Figure S1A). We further analyzed the association between tumor purity and 23 m^6^A modification regulators (Figure 1G) and found that most of the m^6^A regulators were positively correlated with tumor cell purity, whereas WTAP has a negative association, suggesting that WTAP was enriched in non-tumor cell components. Forest plots with Cox regression model were employed to speculate the relationship between m^6^A regulators and the prognosis of melanoma patients. We found that samples with high expression of WTAP, FMR1, and METTL14 were associated with improved overall survival, while an opposite tendency was observed in RBM15B and ELAVL1 (Supplementary Figure S1B). Taken together, we integrated the genomic and transcriptomic landscapes of m^6^A regulators in melanoma, and noticed the changes in the expression levels and genetic variation of m^6^A regulators driving the development and progression of melanoma.

The m^6^A Methylation Modification Pattern Consisting of 23 m^6^A Regulators Is Associated with Prognosis in Melanoma Patients.

**FIGURE 2:**
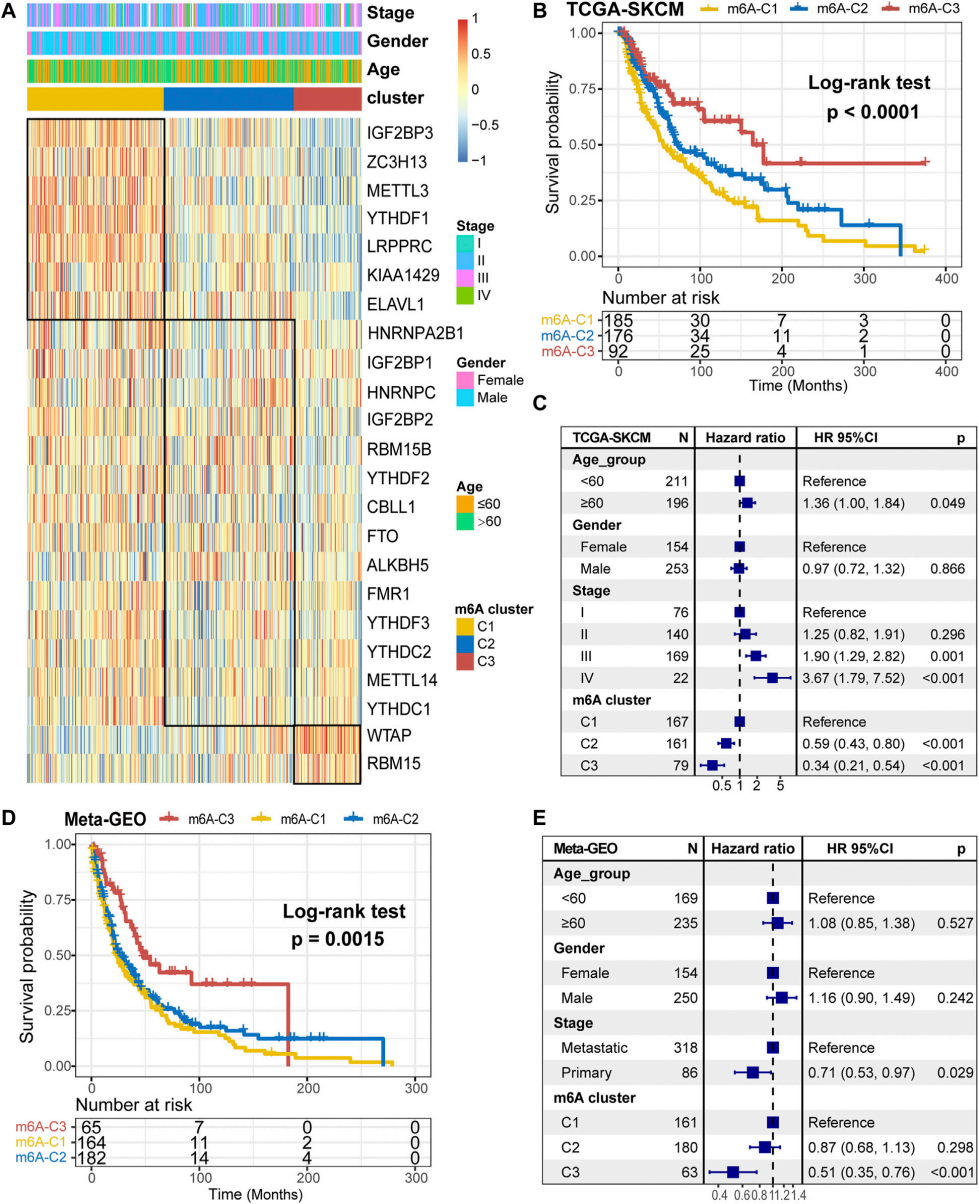
m^6^A methylation modification cluster and unsupervised clustering. **(A)** Results of unsupervised clustering of gene expression of 23 m^6^A moderators in the TCGA-SKCM cohort. **(B)** Kaplan-Meier curves of overall survival (OS) for different m^6^A clusters in the TCGA cohort. **(C)** Subgroup analysis for estimating clinical prognostic value of m^6^A modification subtype after adjusting for age, gender, and stage in the TCGA cohort. **(D)** Kaplan-Meier curves of overall survival (OS) for different m^6^A clusters in the *meta*-GEO cohort. **(E)** Subgroup analysis for estimating clinical prognostic value of m^6^A modification subtype after adjusting for age, gender, and stage in the *meta*-GEO cohort.

Further, we stratified melanoma samples into three m^6^A modification patterns according to the expression of m^6^A regulators, a process based on consensus clustering analysis of the NMF algorithm (Supplementary Figure S2A,C). We named the three clusters as m^6^A-C1, m^6^A-C2, and m^6^A-C3, respectively (Figure 2A). The samples of m^6^A-C2 cluster had significantly different from the other two groups with regard to ELAVL1, RBM15B, YTHDF1/2/3, IGF2BP1/2/3, WTAP, METTL3, ZC3H13, RBM15, HNRNPA2B1, CBLL1, and LRPPRC. Besides, YTHDF1, IGF2BP3, METTL3, ZC3H13, and LRPPRC were significantly upregulated in the m^6^A-C1 subtype, WTAP and RBM15 were significantly upregulated in the m^6^A-C3 subtype, while ELAVL1, IGF2BP3, ZC3H13, and LRPPRC were significantly decreased in the m^6^A-C3 subtype. This conclusion was validated in the *meta*-GEO cohort consisting of five datasets, which include GSE19234, GSE22154, GSE50509, GSE59455, and GSE65904 (Supplementary Figure S2B). Patients in the m^6^A-C3 cluster have a significant survival advantage than other clusters in both TCGA and *meta*-GEO cohort (log-rank test, TCGA: *p* < 0.0001, Figure 2B; *meta*-GEO: *p* = 0.0015, Figure 2D). This model remained significant after multivariate Cox proportional risk regression analysis adjusted for clinicopathological factors of age, gender, and stage (TCGA: HR = 0.34 (0.21–0.51), *p* < 0.001; *meta*-GEO: HR = 0.51 (0.35–0.76), *p* < 0.001; Figures 2C,E).

### Characterization of the Immune Landscape With Three m^6^A Modification Clusters

**FIGURE 3:**
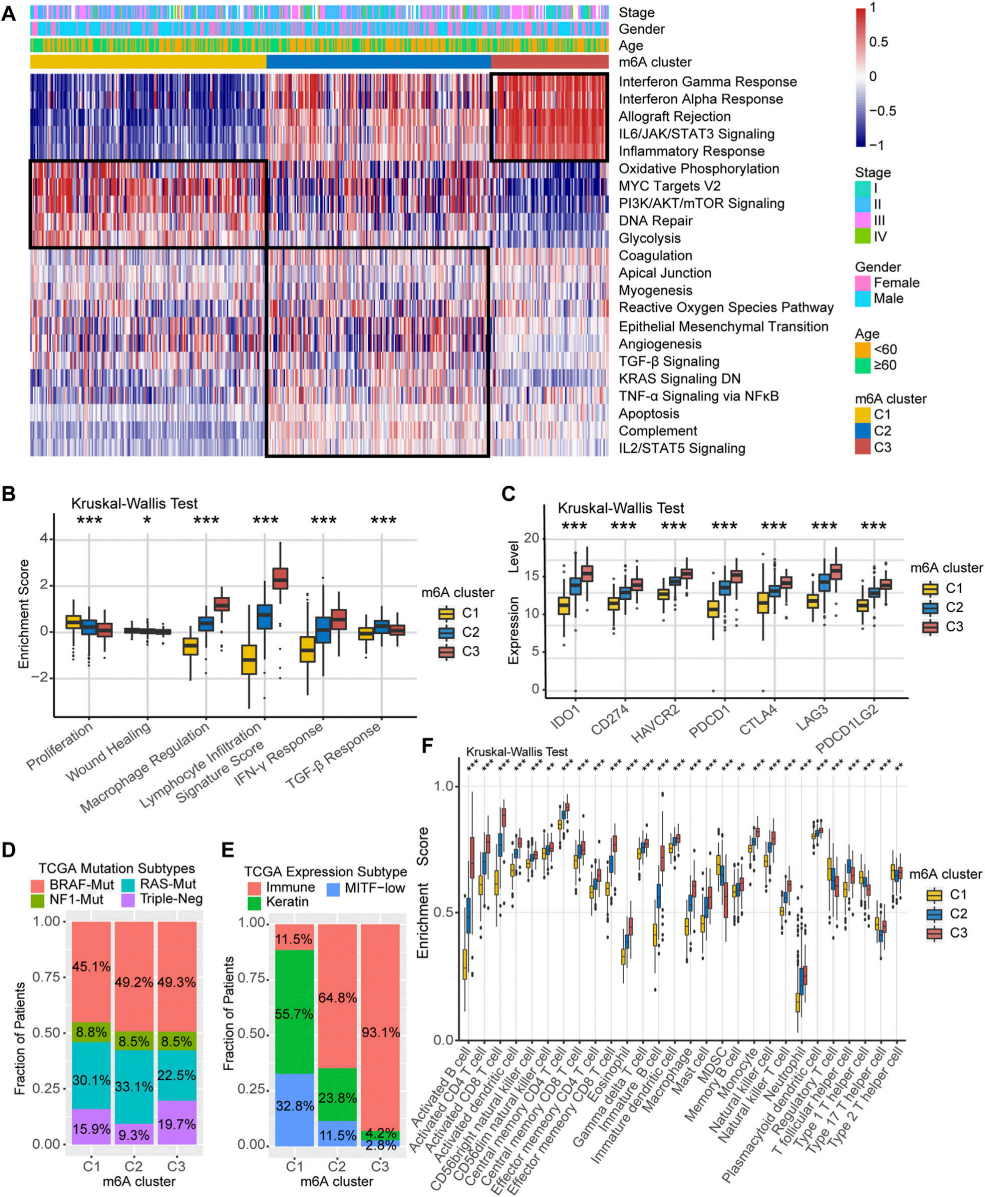
TME characteristics in distinct m^6^A modification clusters. **(A)** Heatmap of enriched pathways based on Hallmark gene set corresponding to different m^6^A modification clusters. **(B)** Relative distribution of six immune subtype in three different m^6^A clusters. **(C)** Expression level of immune checkpoint-related key genes among the three m^6^A clusters. **(D)** Association between TCGA genomic molecular typing and m^6^A clusters. **(E)** Association between TCGA transcriptome molecular typing and m^6^A clusters. **(F)** Relative infiltration level of 28 immune cell subsets among three distinct m^6^A modification clusters.

We performed GSVA analysis against on Hallmarker gene set in an attempt to discover differences in the biological behavior of the three m^6^A modification clusters. As shown in Figure 3A, m^6^A-C1 cluster was associated with cell proliferation and differentiation and glucose transport, including oxidative phosphorylation, PI3K/AKT/mTOR signaling, DNA repair, and glycolysis. m^6^A-C2 cluster is distinguished by cancer and immune surveillance, involving epithelial mesenchymal transition, TGF-β signal, TNF-α signaling *via* NF-κB, and IL2/STAT5 signaling. As for m^6^A-C3, it was significantly enriched in signaling pathways related to inflammation and innate immune response, such as interferon-γ response, interferon-α response, allograft rejection, IL6/JAK/STAT3 signaling, and inflammatory response. The GSVA results further corroborate that these three m^6^A methylation modification clusters are directly related to different molecular mechanism, and m^6^A-C3 was strongly associated with antitumor immunity. In addition, we further evaluated the immune enrichment level of m^6^A methylation modification clusters using the ImmuneScore model constructed by ESTIMATE algorithm. The results showed significant differences in different clusters in both TCGA-SKCM cohort and the *meta*-GEO cohort (Supplementary Figure S3A,B). There is a coherence between the immune activation and survival time, which cluster with higher ImmuneScore having greater survival benefit for patients, like m^6^A-C3 in the TCGA-SKCM cohort and *meta*-GEO cohort. Thorsson et al. (Thorsson et al., 2018) divided the tumor immune landscape into six immune subtypes, represented with immune infiltration and stromal activation. Consistent with our findings, the m^6^A-C1 cluster is more inclined to the “Proliferation” and “Wound Healing” subtypes, m^6^A-C2 is highly expressed in “TGF-β Response,” and m^6^A-C3 is mainly dominated by “Lymphocyte Infiltration Signature Score,” “Macrophage Regulation,” and “IFN-γ Response” subtypes (Figure 3B). In addition, we performed a comparative analysis of immune checkpoint-related key genes (IDO1, CD274, TIM-3, PDCD1, CTLA-4, LAG3, and PDCD1LG2) among the three clusters. The results indicated the expression levels of seven key genes differ significantly between the three clusters, and the highest expression was all observed in the m^6^A-C3 cluster. TCGA-SKCM has established molecular typing based on the genomic landscape and transcriptomic profile. Although the proportion of m^6^A modification clusters among different mutational-based molecular subtype (BRAF-Mut, RAS-Mut, NF1-Mut, and Triple Negative) were not significant (Figure 3D), an obvious difference was found in transcriptomic-based subtype across three m^6^A modification clusters (Figure 3E). Samples with TCGA-Immune subtype account for 93.1% of the m^6^A-C3 cluster, followed by 64.8% in m^6^A-C2 subtype. However, the m^6^A-C1 were dominated by TCGA-Keratin (55.7%) and TCGA-MIFT-low (32.8%) subtype, which demonstrated the desert-related immune phenotype in m^6^A-C1. It is feasible to determine immune cell type abundance and expression from bulk tissues with digital cytometry (Newman et al., 2019). We also compared the immune cell infiltration level among the three m^6^A modification clusters in Figure 3F. The m^6^A-C1 cluster showed more myeloid-derived suppressor cell (MDSC), regulatory T cells, and T helper cell infiltration, while the m^6^A-C3 cluster exhibited infiltration of most types of T cells, natural killer cells, and dendritic cells. This suggested that the lower survival risk of melanoma patients with m^6^A-C3 clusters may be due to effective activation of the pre-existing immunity to inhibit tumor growth and malignant progression. Likewise, evaluation on immune cell abundance by CIBERSORT algorithm also corroborate our conclusions (Supplementary Figure S3C). In addition, the association between each m^6^A regulator and immune cell infiltration was also explored. As expressed in Supplementary Figure S3D, upregulation of WTAP and ALKBH5 was positively correlated with enhanced immune infiltration, while high expression of LRPPRC, METTL3, YTHDF1/3, and ZC3H13 was mostly associated with immunosuppression.

### Differentially Expressed Genes Associated With m^6^A Methylation Modifications in Melanoma

**FIGURE 4:**
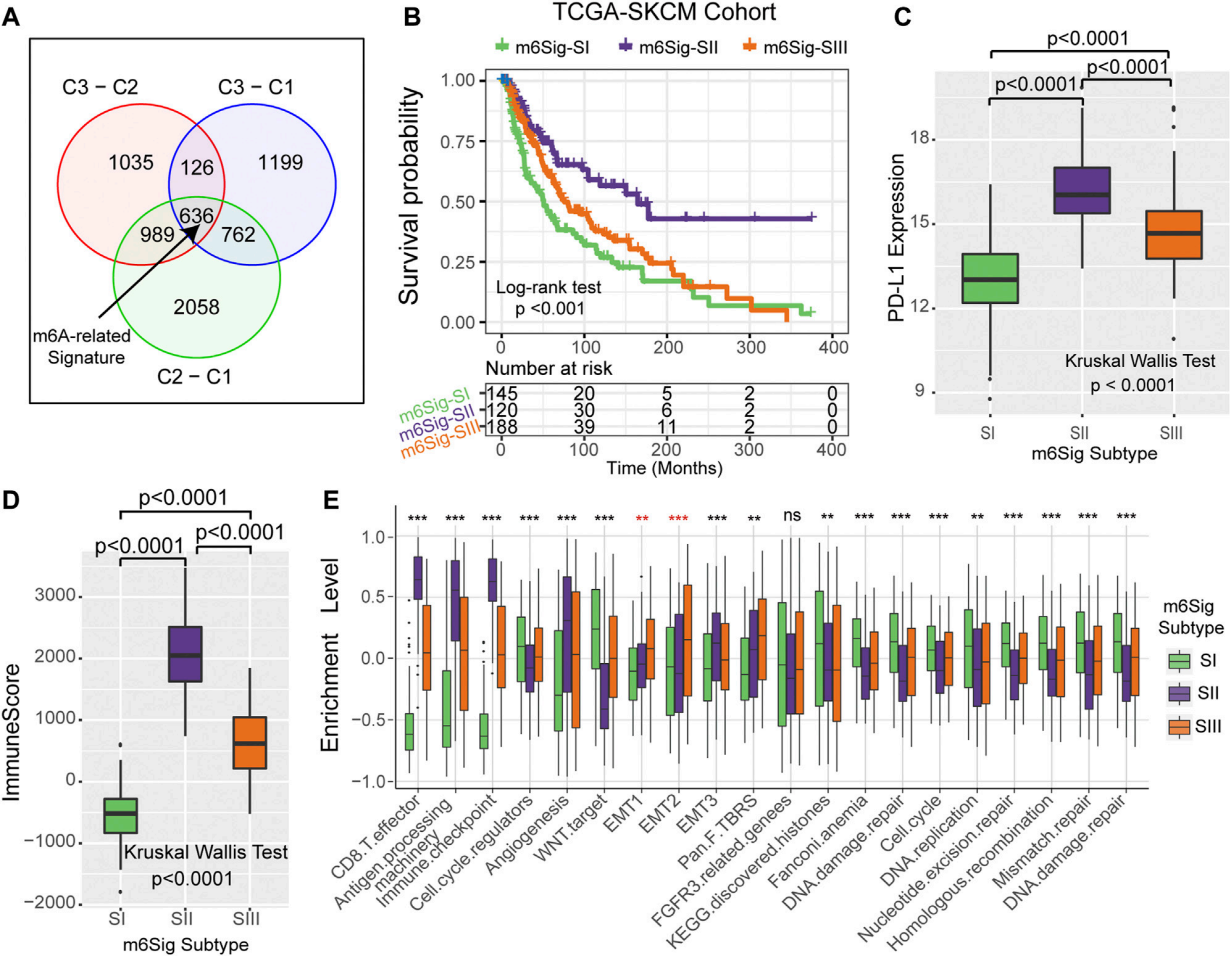
Construction of differential expression of m^6^A gene signatures and functional annotation. **(A)** The 636 differentially expressed genes between the three m^6^A clusters were recognized as m^6^A-related gene signature and shown in the Venn diagram. **(B)** Survival curves of m^6^A signature gene-based NMF unsupervised clustering in TCGA cohort. **(C)** Differences in PD-L1 expression among m^6^Sig subtype groups. **(D)** Differences in ImmuneScore between m^6^sig subtype groups. **(E)** Enrichment level of the three m^6^Sig subtypes in the classical signaling pathway constructed by Mariathasan et al.

Since RNA N6-methyladenosine (m^6^A) modification plays an important role in post-transcriptional regulation, we further examine the potential impact on gene expression change of each m^6^A modification cluster in melanoma. To clarify these queries, we employed the Bayesian-based method to identify differentially expressed genes (DEGs) that are differentially regulated across the three m^6^A methylation modification clusters. As illustrated in the Venn diagram of Figure 4A, there are a total of 636 DEGs which may play the crucial role in distinguishing the three m^6^A modification clusters (Supplementary Table S3). Based on these 636 representative DEGs of m^6^A signature, we further stratified the melanoma samples into three well typed transcriptomic phenotypes (denoted as m^6^Sig-SI, m^6^Sig-SII, and m^6^Sig-SIII) by unsupervised consensus clustering analysis (Supplementary Figure S4A). We also compared the m6A clusters and m6A signature-derived subtype, and found a significant association among these two-stratification method (Supplementary Table S4, adjusted χ^2^ test, *p* < 0.0001). Patients in m^6^Sig-SII were proved to be associated to better prognosis, while m^6^Sig-SI had a worse outcome (*p* < 0.001, log-rank test; Figure 4B). PD-L1 and ImmmuneScore were also highly expressed in m^6^Sig-SII subtype than the other subtypes (*p* < 0.0001, Kruskal Wallis test; Figures 4C,D). Mariathasan et al. (Mariathasan et al., 2018) summarized and formed a suit of gene set for assessing the activation of immune and stroma signaling pathway, whereby we adopted to evaluate the m^6^Sig signature. The m^6^Sig-SI subgroup was enriched in cell proliferation and DNA damage repair related pathways, m^6^Sig-SIII was characterized by CD8 T effector and antigen processing related pathway, whereas m^6^Sig-SII was focused in epithelial mesenchymal transition (EMT) related signaling pathway (Figure 4E). The expression level of 23 m^6^A regulators in three gene subgroups was also compared and shown in Supplementary Figure S4B. We observed significant differences of m^6^A regulator expression in the three m^6^A gene-signature subgroups, which was consistent with the expected results of the m^6^A methylation modification clusters.

### The m^6^Sig Score System and Its Clinical Relevance

**FIGURE 5:**
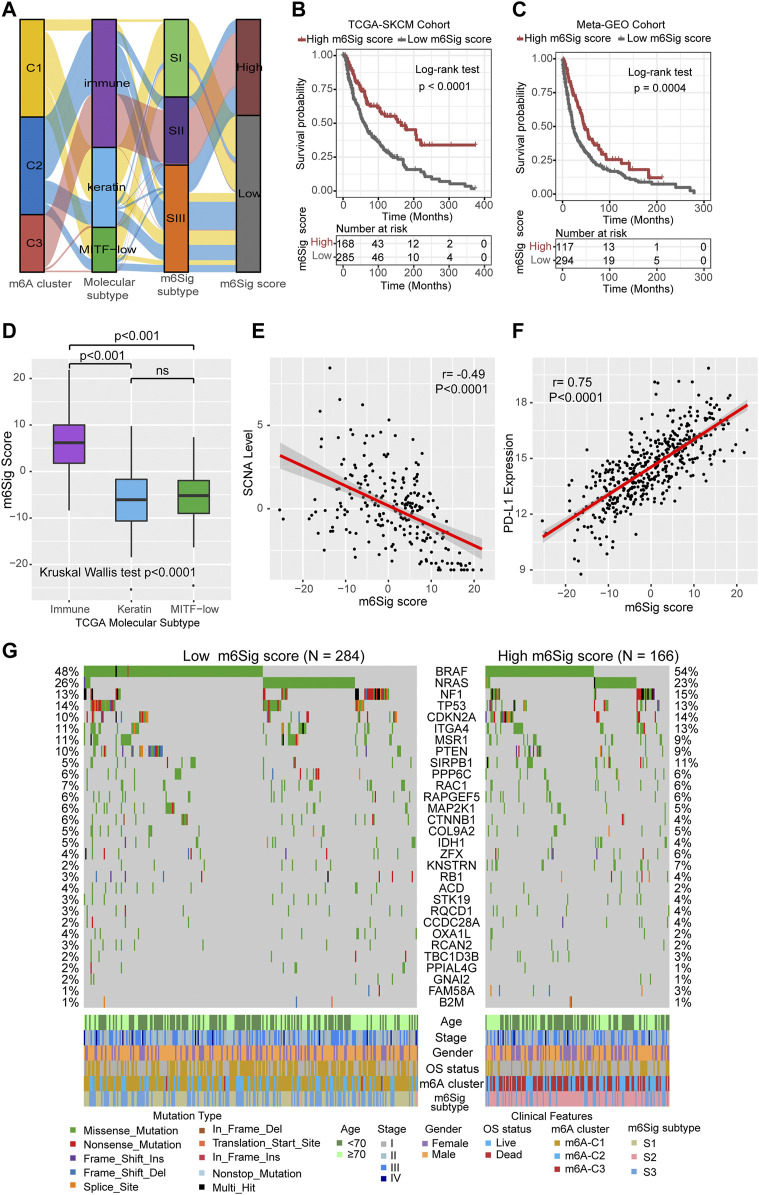
Construction of m^6^Sig score and explore the relevance of clinical features. **(A)** Alluvial diagram of m^6^A clusters in groups with different molecular subtypes (immune, keratin, and MITF-low), m^6^A-gene cluster, and m^6^Sig score. **(B)** Kaplan-Meier curves for high and low m^6^Sig score patient groups in TCGA cohort. **(C)** Kaplan-Meier curves for high and low m^6^Sig score patient groups in *meta*-GEO cohort. **(D)** The m^6^Sig score differed between the three TCGA molecular types. **(E)** The m^6^A score was negatively correlated with the SCNA mutational level. **(F)** The m^6^A score was positively correlated with PD-L1 expression level. **(G)** Mutation status of significantly mutated genes (SMGs) in the TCGA cohort, stratified by subgroups with low (left) versus high m^6^Sig scores (right). Each column represents one patient. Mutation types and clinical characteristics were annotated in different colors.

The results of the previous parts of the study can be concluded that m^6^A methylation modification has a strong association with prognosis and immune regulation in melanoma patients. In order to be able to accurately predict the m^6^A methylation modification cluster of tumors in individual melanoma patients, we developed the m^6^Sig score system to quantify the m^6^A modification cluster based on the identified m^6^A-related signature genes. In Figure 5A, the Sankey diagram uncovered the workflow of the m^6^Sig score system in melanoma. It can be found that m^6^A-C3 was linked to a higher m^6^Sig score, and lower for keratin and MITF-low subtypes. We sought to evaluate the significance of the m^6^Sig score system in predicting the prognosis of patients with melanoma through survival analysis. As shown in Figures 5B,C, the m^6^Sig score system clearly distinguished patient with different prognosis in both the TCGA-SKCM cohort and *meta*-GEO cohort (patients with high m^6^Sig scores had a better prognosis). We performed the Kruskal Wallis test and showed that the m^6^Sig score could be clearly distinguished in the immune and keratin subtypes (Figure 5D). Encouragingly, the m^6^Sig score system can be extremely well distinguished among the previous m^6^A clusters and m^6^Sig clusters (Supplementary Figure S5A,B), which are clusters constructed based on m^6^A methylation modification clusters and DEGs, respectively. Compared with clinicopathological staging, the m^6^Sig score system can better evaluate the prognosis of melanoma patients (AUCs for stage and m^6^Sig score are 0.613 and 0.681, respectively; Supplementary Figure S5C). In detail, multivariable cox regression model revealed that melanoma patients with low m^6^Sig scores had a worse survival outcome in TCGA (Supplementary Figure S5D) and *meta*-GEO cohort (Supplementary Figure S5E). This score system was also explored by ImmuneScore, and patients with high m^6^Sig scores had a higher ImmuneScore (*p* < 0.0001, Supplementary Figure S5F,G). Among the six immune subtypes, the m^6^Sig score was also differentially distributed. Samples with high scores were mostly clustered in the “Macrophage Regulation,” “Lymphocyte Infiltration Signature Score,” and “IFN-γ Response” subtypes (Supplementary Figure S5H), which was similar to the m^6^A-C3 cluster. Heatmap of correlation matrix demonstrated that the m^6^Sig score was markedly positively correlated with the immune activation process and negatively correlated with cell cycle and DNA damage repair (Supplementary Figure S5I). To better evaluate the effectiveness of our m^6^Sig score system in predicting the prognosis of melanoma patients, we introduced two independent cohorts (GSE22153 and GSE54437) to perform a survival analysis, and the results showed that patients with high m^6^Sig score had a better prognosis (Supplementary Figure S6A,B). Furthermore, a survival analysis after combining all patients involved in this study revealed that a high m^6^Sig score continued to indicate a survival benefit in melanoma patients (*p* < 0.0001, Supplementary Figure S6C). The TCGA-SKCM cohort also demonstrated that melanoma patients with high m^6^Sig score had prolonged disease-free survival (DFS, *p* = 0.0064, Supplementary Figure S6D). In addition, m^6^Sig score also negatively correlated with somatic copy number alternation (SCNA) level (*r* = −0.49, *p* < 0.0001, Figure 5E), which is a significant predictor of immunotherapy resistance in melanoma. The PD-L1 expression levels were also positively correlated with the m^6^Sig score (*r* = 0.75, *p* < 0.0001, Figure 5F), suggesting that melanoma patients may also benefit from the m^6^Sig score system for PD1/PD-L1 treatment regimens. More deeply, we performed significant mutation gene (SMG) analysis of melanoma samples based on m^6^Sig score, and the waterfall plot of mutation landscape noted that BRAF (54%/48%), SIRPB1(11%/5%), and KNSTRN (7%/2%) had higher somatic mutation rates in the high-score group, although BRAF was not statistically significant (Figure 5G). These data assist us to more comprehensively understand the m^6^Sig score system mapping to genomic variants, predicting that m^6^A methylation modification is closely linked to somatic mutations in melanoma patients.

The m^6^Sig Score System Can Be a Better Predictor of the Effectiveness of Immunotherapy in Cancer

**FIGURE 6:**
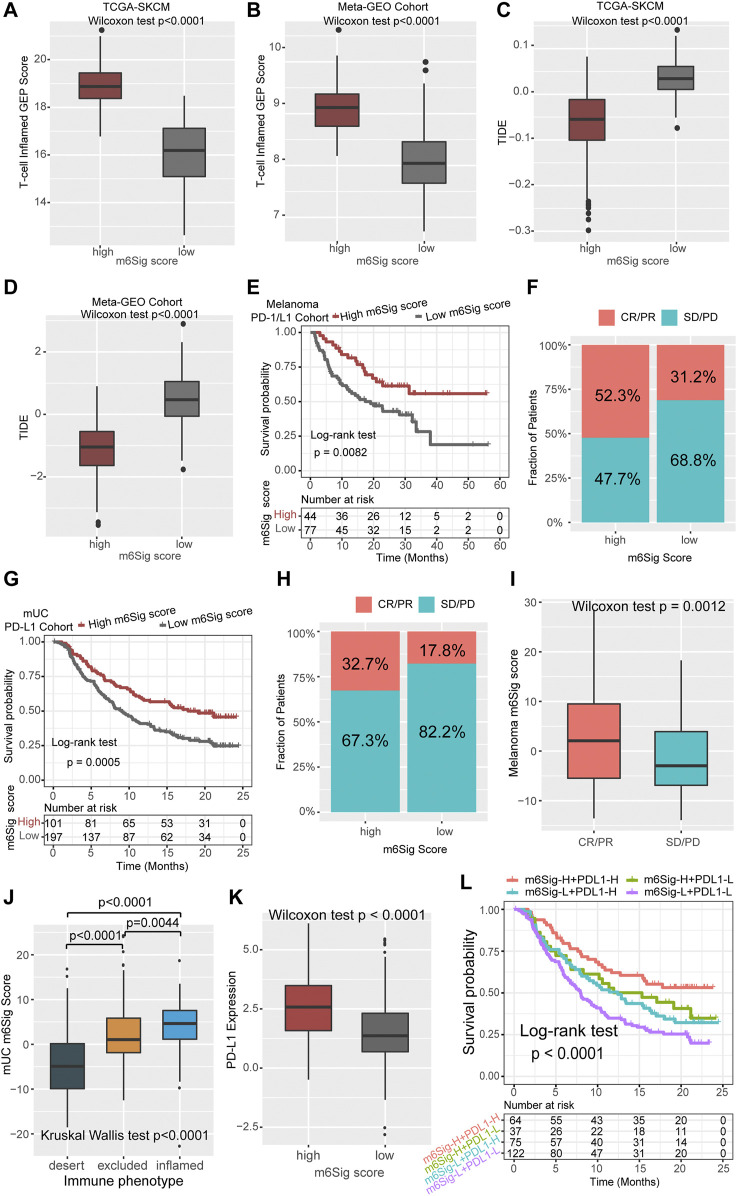
The m^6^Sig score predicts immunotherapeutic benefits. **(A)** Comparison of the relative distribution of T-cell inflamed GEP scores between the high and low m^6^Sig score groups in the TCGA cohort. **(B)** Comparison of the relative distribution of T-cell inflamed GEP scores between the high and low m^6^Sig score groups in the *meta*-GEO cohort. **(C)** Comparison of the relative distribution of TIDE between the high and low m^6^Sig score groups in the TCGA cohort. **(D)** Comparison of the relative distribution of TIDE between the high and low m^6^Sig score groups in the *meta*-GEO cohort. **(E)** Kaplan-Meier curves for high and low m^6^Sig score patient groups in the melanoma PD-1/CTLA-4 cohort. **(F)** The fraction of patients with clinical response to anti-PD-1/CTLA-4 immunotherapy in low or high m^6^Sig score groups. **(G)** Kaplan-Meier curves for high and low m^6^Sig score patient groups in the metastatic urothelial carcinoma (mUC) PD-L1 cohort. **(H)** The fraction of patients with clinical response to anti-PD-L1 immunotherapy in low or high m^6^Sig score groups of mUC cohort. **(I)** Distribution of m^6^Sig scores between immunotherapy response and non-response in melanoma PD-1/CTLA-4 cohort. **(J)** Distribution of mUC m^6^Sig scores among the three immune phenotypes. **(K)** The relationship between m^6^Sig score and PD-L1 expression level. **(L)** The m^6^Sig score combined with PD-L1 expression levels better predicted patient prognosis. CR, complete response; PR, partial response; SD, stable disease; PD, progressive disease.

Cancer treatment regimens based on immune checkpoint inhibitors have provided a landmark innovation in the treatment of malignancies, mostly in melanoma. In addition to TML and PD-L1, TIDE and T cell-inflamed GEP have been recommended to predict immune response in recent years (Chen et al., 2019c; Chen et al., 2019d). We compared the established m^6^Sig score system with the T-cell inflamed gene expression profile (GEP) score and found that melanoma patients with high m^6^Sig score had elevated T-cell inflamed GEP score in both the TCGA-SKCM cohort and the *Meta*-GEO cohort (*p* < 0.0001, Figures 6A,B). In contrast, TIDE showed increased levels in patients with low m^6^Sig scores, implying that greater chance of tumor immune escape and resistance in low m^6^Sig scores subgroup (*p* < 0.0001, Figures 6C,D). These results further demonstrate that m^6^A modification clusters play a critical role in the immune response of tumors, thereby affecting the immune microenvironment of tumors.

Aforementioned data point to a strong association between m^6^A modification and immune response, we next investigated whether the m^6^Sig score could predict patients’ response to ICI treatment in independent immunotherapy cohorts. Patients with high m^6^Sig score exhibited significantly longer survival time (log-rank test, *p* = 0.0082, Figure 6E) and markedly clinical response to PD-1/CTLA-4 treatment in melanoma (response rate, high vs low m^6^Sig score subgroup, 52.3 vs 31.2%, Figure 6F). This result was also identified in an anti-PD-L1 metastatic uroepithelial cancer cohort (Mariathasan et al., 2018), in which patients with high m^6^Sig scores significantly benefited from PD-L1 immunotherapy (log-rank test, *p* = 0.0005, Figure 6G; response rate: high vs low m6Sig score subgroup, 32.7 vs 17.8%, Figure 6H). Furthermore, we found that patients with PD-1 immune response also had a higher m^6^Sig score (*p* = 0.0012, Figure 6I). Metastatic uroepithelial carcinoma patients with immune inflamed phenotype had a higher m^6^Sig score than immune excluded and desert phenotype (Figure 6J). A significant elevation of PD-L1 was identified in high m^6^Sig score subgroup (*p* < 0.0001, Figure 6K). Therefore, we divided the overall population into four subgroups according to the TMEsig-score and PD-L1 distribution, including TMEsig-score-H + PD-L1-H, TMEsig-score-H + PD-L1-L, TMEsig-score-L + PD-L1-H, and TMEsig-score-L + PD-L1-L. The TMEsig-score-H + PD-L1-H subgroup exhibited the best prognostic outcome compared with the other three subgroups (log-rank test, *p* < 0.0001, Figure 6L). Based on the results of the above analysis, our established m^6^Sig score system enables the prediction of responsiveness and prognosis to cancer immunotherapy.

## Discussion

Recently, the dynamic and reversible process of m^6^A modification has been reported in participation of the innate immune, inflammatory response, and anti-tumor processes (Chen et al., 2019a; Shulman and Stern-Ginossar, 2020). Although numerous studies have recently revealed how m^6^A regulators are epigenetically regulated in the tumor immunogenicity, the association between m^6^A regulators and the overall tumor microenvironment has not yet been elucidated in melanoma. Thus, identifying distinct m^6^A modification clusters in the TME infiltration will contribute to advancing our understanding of anti-tumor immune response and facilitating more effective precision immunotherapy strategies.

In this study, we identified three different immunophenotypic m^6^A methylation modification clusters, which are characterized by different anticancer immune effects. The m^6^A-C1 phenotype is distinguished by promotion of cell proliferation and activation of PI3K/AKT/mTOR signaling pathway, and we prefer it to be the immune-desert phenotype. The m^6^A-C2 phenotype is more characteristic of cancer and immune surveillance, and it is associated with EMT, TGF-β, and TNF-α pathway activation and is an immune-excluded phenotype. The m^6^A-C3 phenotype, on the other hand, is associated with activation of pathways related to inflammatory response, innate immune response, and is an immune-inflamed phenotype. It has been shown that the tumor microenvironment plays a central role in tumorigenesis development and progression, and the levels of tumor-infiltrating CD4^+^/CD8^+^ T cells, M1 macrophages, NK cells, and inflammatory cytokines directly influence the onset of immune priming and adaptive immunity (Topalian et al., 2016; Galon and Bruni, 2019; Zeng et al., 2020). Interestingly, the m^6^A-C2 phenotype is associated with activation of the TGF-β signaling pathway and intermediate immune cell infiltration, and thus, we hypothesized that melanoma patients with m^6^A-C2 phenotype would benefit from the combination of immune checkpoint inhibitors and TGF-β blockers. There is evidence pointing out that activation of the TGF-β pathway hinders lymphocyte attack on “tumor barriers” (Tauriello et al., 2018). Moreover, inhibitors targeting TGF-β can effectively remodel the tumor microenvironment in the form of reprogrammed peritumor stromal fibroblasts, which can restore the body’s anti-tumor immunity (Mariathasan et al., 2018; Panagi et al., 2020). We found that the m^6^A-C3 phenotype was associated with enrichment of activated tumor-infiltrating lymphocytes, making this phenotype more likely responsive to ICI immunotherapy.

The m^6^A signature genes were derived from genes differentially expressed in three m^6^A modification subtypes. These m^6^A-related signature genes were further utilized to identify transcriptomic subtypes and tumor microenvironment landscapes in melanoma. Patients with m^6^Sig-SII subtype have higher PD-L1 expression levels and higher immuneScores, implying that patients with this subtype are better treated with immune checkpoint inhibitors for better therapeutic outcomes. For the sake of precision clinical practice, we optimized the m^6^Sig signatures into the m^6^Sig score scheme, a system that could be used to quantify the m^6^A modification level of individual tumors. The m^6^A modification clusters characterized by an immune-inflamed phenotype showed a higher m^6^Sig score, whereas the modification cluster characterized by an immune-desert phenotype had a lower score. The results based on survival analysis highlight that the m^6^Sig score system can effectively predict the prognosis of melanoma patients, and that this score system is strongly associated with TCGA molecular subtypes, genomic alternations, and PD-L1 expression levels. We also observed that m6Sig score was closely correlated with T-cell inflamed GEP score and TIDE, which are effective tools for prediction of immunotherapy benefit, further demonstrating that m^6^A RNA methylation modification can modulate the effect of immune response in melanoma. To identify the predictive value of m^6^Sig score system in immune response, we performed a series of analyses in two additional independent immunotherapy cohorts and validated the effect of this score system. In a nutshell, the m^6^A RNA methylation modification cluster can be used to determine the immune phenotype of melanoma patients, further guiding clinical treatment planning and effectively predicting the prognosis of patients.

We also noticed that certain m^6^A regulators play different roles in regulating tumorigenesis and tumor immunogenicity. Recent studies have confirmed that the mRNA stability and translation processes of the oncofetal IGF2 mRNA binding proteins (IGF2BPs) are regulated by RNA N6-methyladenosine (Huang et al., 2018). IGF2BP1, a member of the IGF2BPs family, was then identified as an oncogene that promotes cancer development by antagonizing cancer-suppressive miRNAs (Müller et al., 2018; Müller et al., 2019). In contrast, our results showed that IGF2BPs genes have higher expression level in patients with metastatic melanoma and m^6^A-C1 subtype. It has been demonstrated that IGF2BP2 promotes cancer progression by regulating the m^6^A-dependent glycolytic process and promotes cancer metastasis in the form of an RNA-protein ternary complex (Chen et al., 2019e). KIAA1429 is also well known as an m^6^A methyltransferase. In hepatocellular carcinoma, KIAA1429 promotes cancer metastasis and leads to poor patient prognosis by regulating post-transcriptional modifications (Lan et al., 2019). Our results also suggest this function of KIAA1429 to promote metastasis and highly expressed in m^6^A-C1 desert phenotype, but its prediction of patient survival may require the combination of RBM15, RBM15B, IGF2BP3, and HNRNPA2B1, with co-occurrence between them. YTHDCs and YTHDFs containing YTH domain act as “readers” in post-translational RNA methylation modification, and YTHDFs enhance aerobic glycolysis by degrading mRNA to further promote tumor formation (Wang et al., 2021; Xia et al., 2021). Our study confirms that both YTHDF1/3 and YTHDC1/2 are highly expressed in metastatic melanoma, and of interest, the high expression of YTHDF1 in patients with metastatic melanoma is accompanied by an indication of a poorer prognosis, which suggests a new direction for deeper studies of molecules containing YTH domain. In our study, we found that ELAVL1 was not only associated with metastasis of melanoma, but also reflected a poorer prognosis of patients, which may be related to the fact that ELAVL1 can stabilize oncogenic transcripts (Li et al., 2020b). In summary, the results of our analysis demonstrate the importance of a systematic and comprehensive consideration of m^6^A modification clusters, which are diverse in cancer across physiological processes.

Identification of significantly mutated genes underlying human cancers is a critical foundation for cancer diagnostics, therapeutics, and selection of rational therapies. In our study, we found a higher proportion of SMGs of BRAF, SIRPB1, and KNSTRN in the high m^6^Sig score subgroup, although BRAF was of marginal significance. In a pan-cancer study, it was noted that BRAF has a higher rate of specific driver mutations in leukocytes of cancer patients, a phenomenon associated with tumor-immune cell interactions (Thorsson et al., 2018). There is new evidence that BRAFi induces the occurrence of anti-tumor cell scorching immune responses, which may be a new strategy for the treatment of melanoma (Erkes et al., 2020). Signal regulatory protein beta 1 (SIRPB1) is a member of the signal regulatory protein (SIRP) family, which also belongs to the immunoglobulin superfamily, and is a negatively regulated receptor-type transmembrane glycoprotein involved in receptor tyrosine kinase-coupled signaling processes. SIRPB1 is associated with neutrophil migration across the epithelium, which provides a new target for drug design in immunotherapy (Ribeiro et al., 2019). It has been reported that KNSTRN mutations rarely occurred in other solid tumors and leukemias, which are relatively specific for skin-related cancers (Lee et al., 2016; Schmitz et al., 2019). These tumor driver mutations in different m^6^Sig scores not only are associated with malignant progression, metastasis, and recurrence of cancer but also play a role in the regulation of immune activity, demonstrating a complex and consequently clear interaction between m^6^A RNA methylation modifications and tumor immunogenomic.

The literature review helped us to integrate the well-known 23 m^6^A RNA methylation regulators for meta-analysis, but this still requires newly discovered regulators to be included to enhance the accuracy of the established m^6^A modification clusters. There is a relative lack of PD-L1-based regimens for melanoma patients, so we introduced a dataset of uroepithelial carcinoma treated with atezolizumab, but we still hope that the m^6^Sig score system can be analyzed and validated in melanoma immunotherapy with different immune checkpoint inhibitors. Moreover, all the data in this study were obtained from retrospective cohort, which would introduce some bias. Therefore, our next study focused on establishing a prospective cohort of melanoma patients with immune checkpoint inhibitors to validate and optimize the m^6^Sig score system. In addition, the current m^6^Sig score system does not yet incorporate the clinicopathological characteristics of the patients, which also lead to the drawbacks of the system.

In our study, we systematically assessed the m^6^A modification clusters of 1,020 melanoma patients and comprehensively analyzed the impact of m^6^A modification clusters generated by 23 m^6^A regulators on the cellular infiltration characteristics of the tumor microenvironment. The results of this integrative analysis confirm that RNA methylation is essential for the regulation of tumor immune response, and assessing the m^6^A modification clusters of patient tumors will help us better understand the immune microenvironment infiltration characteristics and provide new ideas for indications and protocol modifications for immunotherapy.

## Data Availability

Publicly available datasets were analyzed in this study. This data can be found here: Gene expression data and clinical information for melanoma patient samples were obtained from the GEO database (https://www.ncbi.nlm.nih.gov/geo/) and TCGA database (https://portal.gdc.cancer.gov/), which are publicly and freely available, including the GSE19234, GSE22154, GSE50509, GSE59455, GSE65904, GSE22153, GSE54437 and TCGA-SCKM datasets.
