# Supplementary material for: Identification of m6A Regulator-Associated Methylation Modification Clusters and Immune Profiles in Melanoma
Source: Front Cell Dev Biol. 2021 Dec 21;9:761134. doi: 10.3389/fcell.2021.761134 (PMC8724425; doi:10.3389/fcell.2021.761134)
Supplement: Supplementary file 1 [file DataSheet1.docx]

Supplementary Material

# Supplementary Figures and Tables

## Supplementary Figures


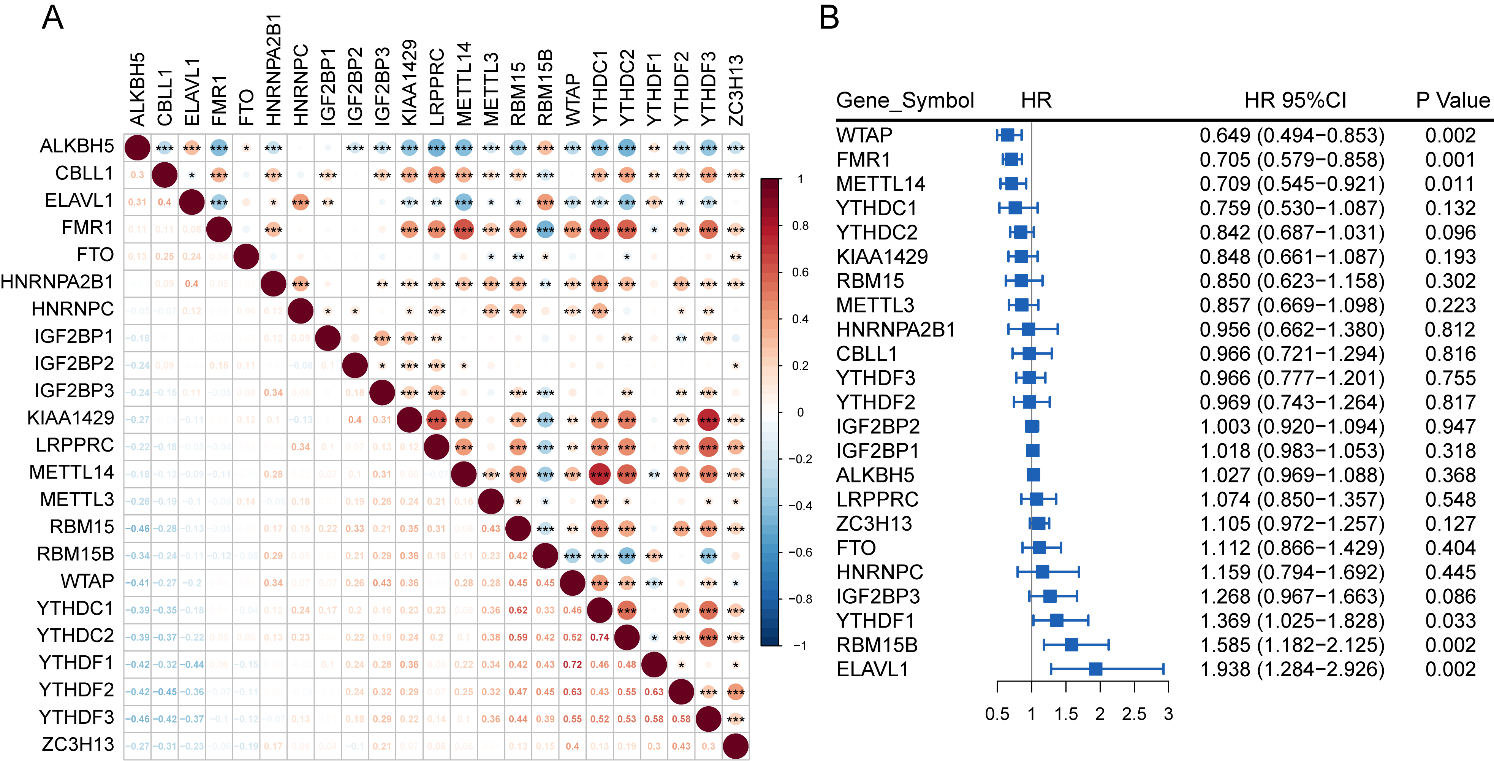


**Supplementary Figure S1.** **Correlation between 23 m^6^A RNA methylation regulators and prognostic analysis in melanoma patients.** (A) Spearman correlation analysis of 23 m^6^A regulators in melanoma patients. Red is a positive correlation and blue is a negative correlation. (B) Cox regression analysis to evaluate the effect of 23 m^6^A modulators on the prognosis of melanoma patients.


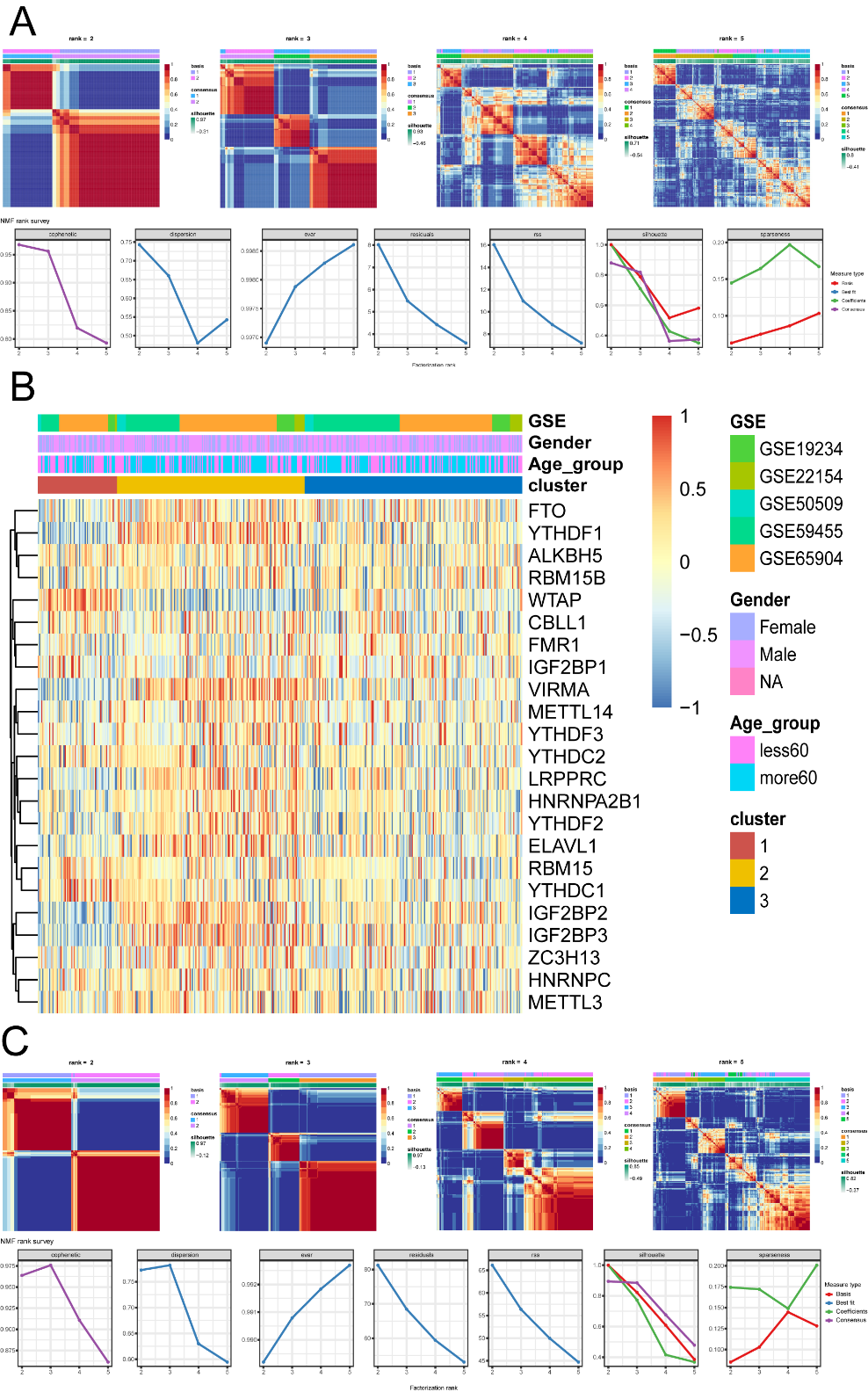


**Supplementary Figure S2.** **Unsupervised clustering of 23 m^6^A regulators in the meta-GEO and TCGA cohorts.** (A) Heatmap of NMF clustering for 23 m^6^A regulators with TCGA database. The cluster numbers range from 2 to 5. (B) Results of unsupervised clustering of 23 m^6^A regulators in melanoma patients from the meta-GEO cohort. (C) Heatmap of NMF clustering for 23 m^6^A regulators with meta-GEO database. The cluster numbers range from 2 to 5.


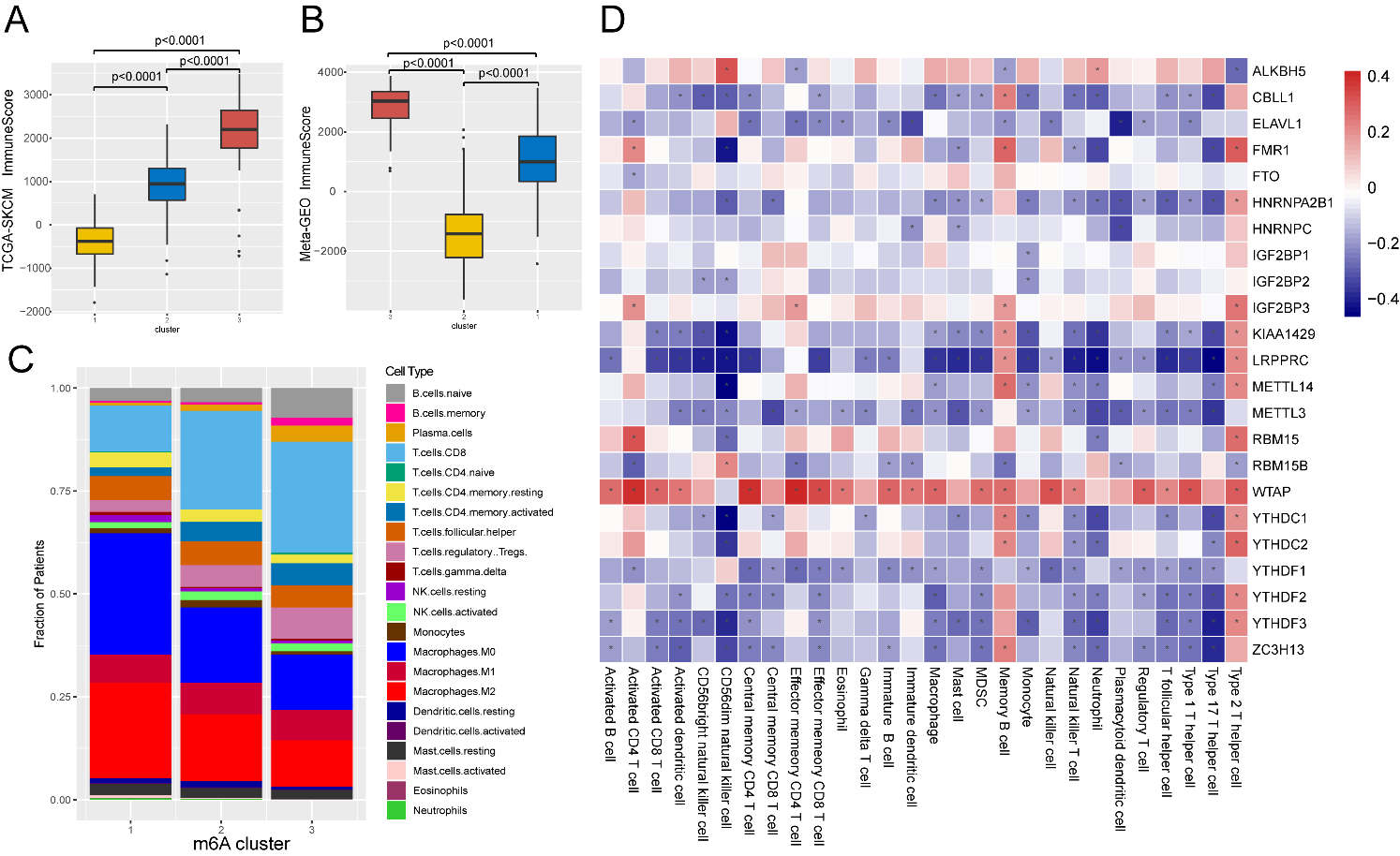


**Supplementary Figure S3.** **The m^6^A regulators are associated with immune activation.** (A) Difference between ImmuneScore of m^6^A clusters in TCGA cohort. (B) Difference between ImmuneScore of m^6^A clusters in meta-GEO cohort. (C) Cellular differences in immune cell infiltration between the three m^6^A clusters were imputed by CIBERSORT. (D) Heatmap of the correlation between 23 m^6^A regulators and Charoentong *et al.* contructed immune cell types. Red is positive correlation and blue is negative correlation.


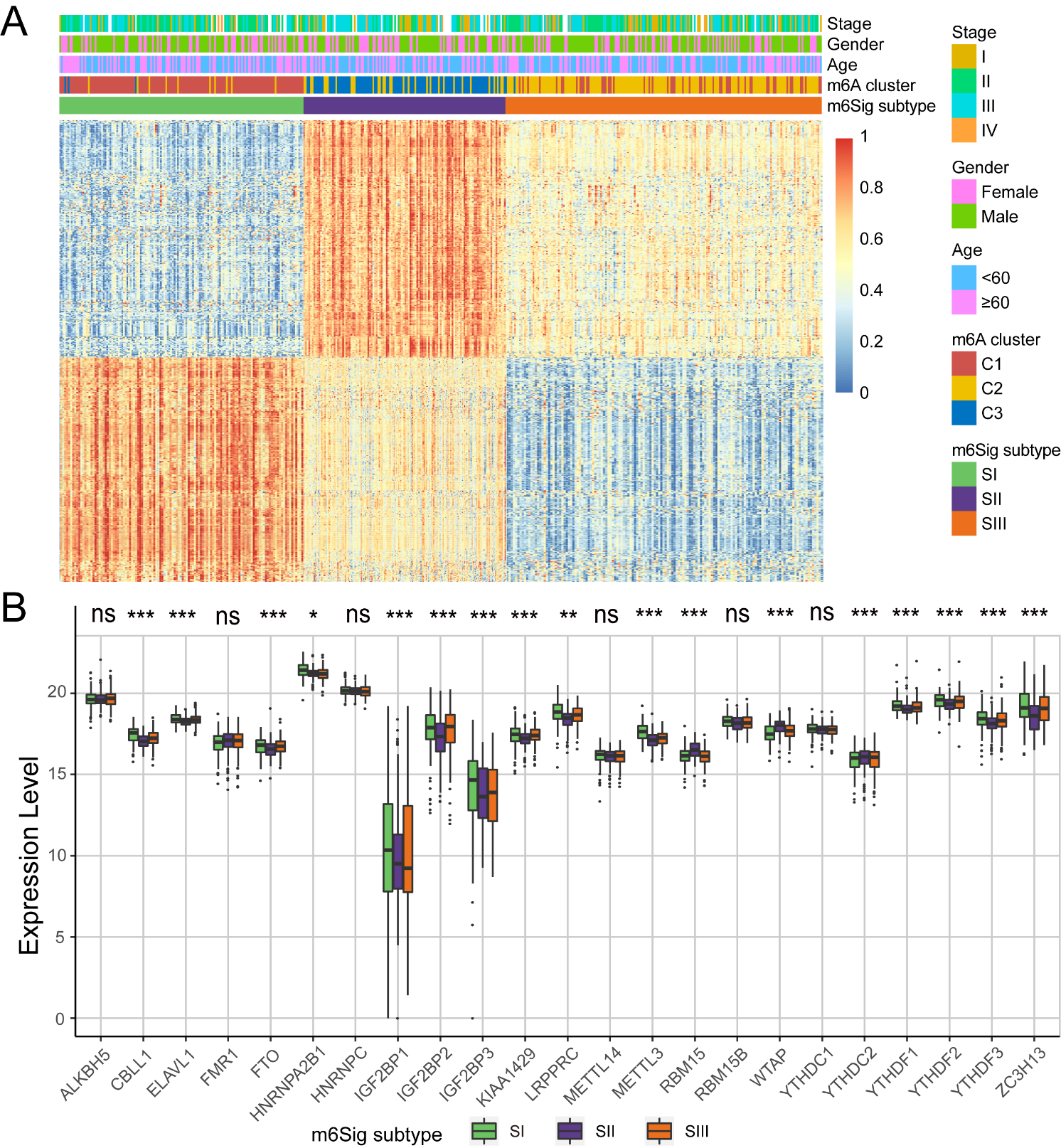


**Supplementary Figure S4.** **Unsupervised clustering representation of m^6^A-related signature genes and association with m^6^A regulators in the melanoma.** (A) Heatmap of 636 m^6^A methylation-associated differentially expressed genes forming m6Sig subtypes. (B) Distribution of expressions of m^6^A regulators among m6A gene-related signatures subtypes.


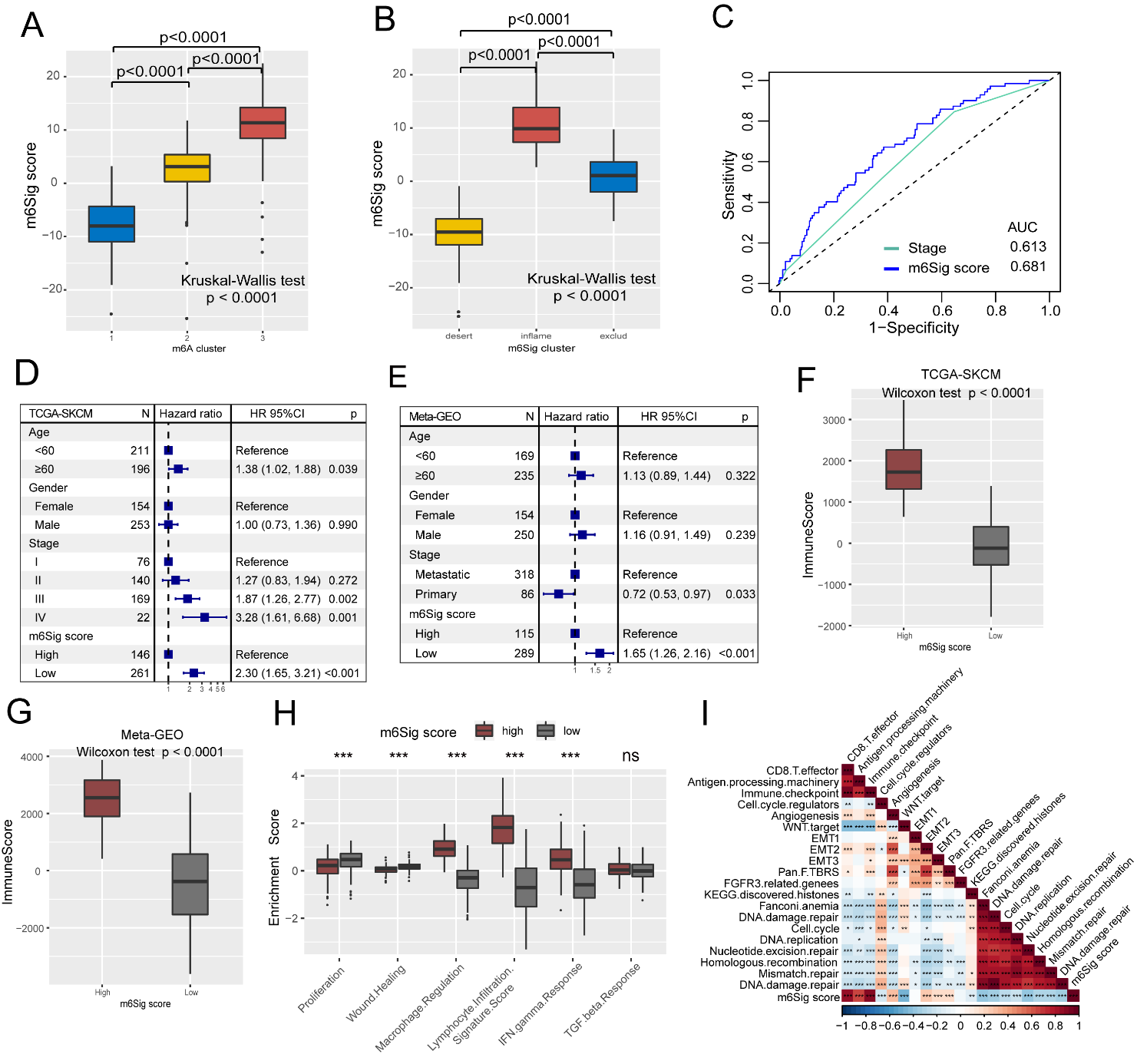


**Supplementary Figure S5.** **m^6^A score associated with TIM immune regulation and survival outcome** (A-B) The TCGA cohort and meta-GEO cohort showed differences in m6Sig scores between different m^6^A clusters. (C) The m6Sig score can effectively predict the prognosis of melanoma patients than stage. (D) Multifactorial Cox regression analysis showed that m6Sig scores were recognized as independent prognostic factor in the TCGA and meta-GEO cohort. (F-G) ImmuneScore differed between m6Sig score subgroups in the TCGA and meta-GEO cohort. (H) Differences in m6Sig scores were correlated with immune activation-associated subtypes. (I) Correlation analysis between m6Sig scores and cancer immune-related biological processes.


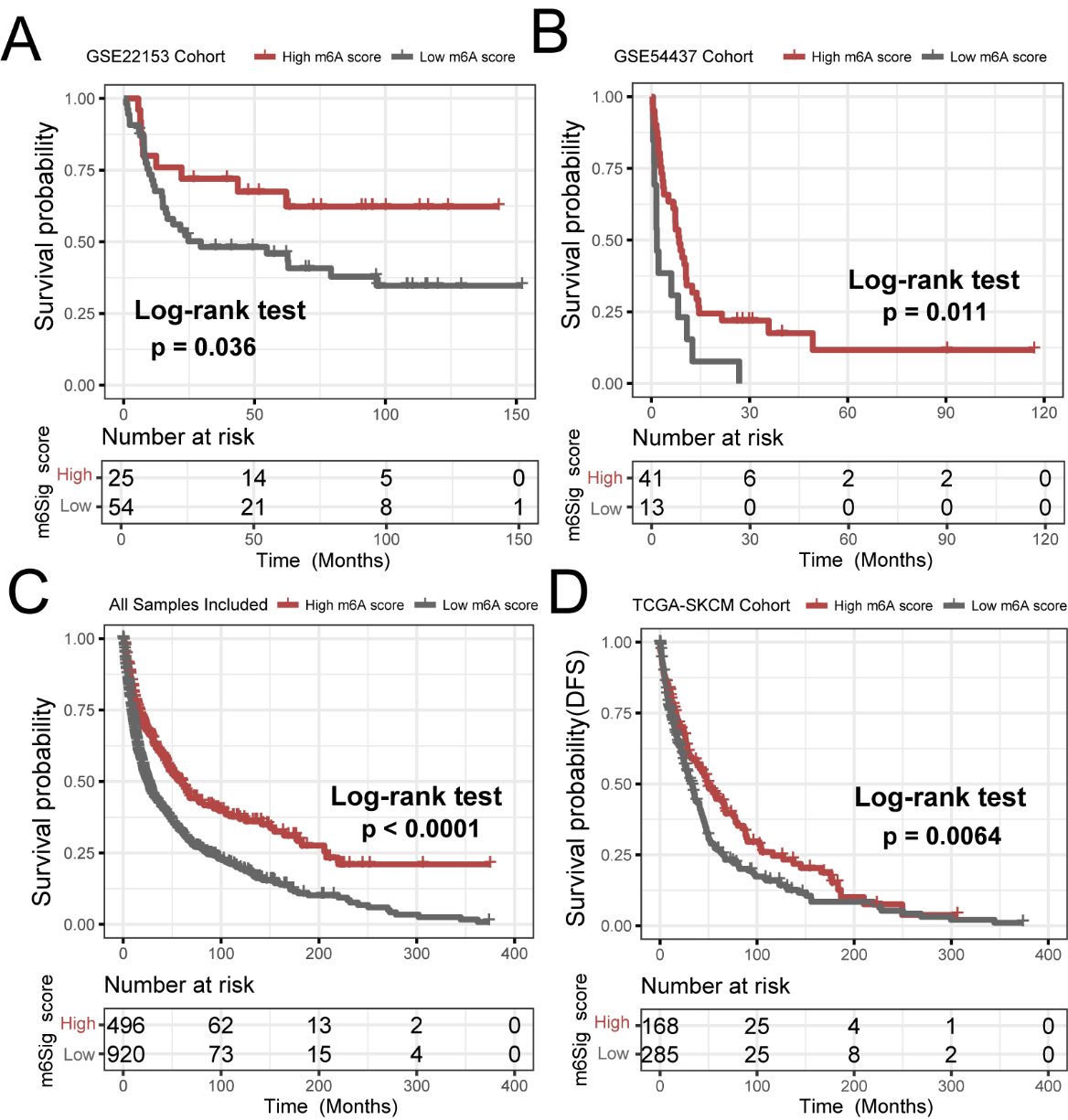


**Supplementary Figure S6.** **The m6Sig score correlates with the prognosis of melanoma patients.** (A) The GSE22153 cohort confirmed that a better prognosis for melanoma patients with higher m6Sig scores. (B) The GSE54437 cohort confirmed that a better prognosis for melanoma patients with higher m6Sig scores. (D) All samples involved in this study confirmed that a better prognosis for melanoma patients with higher m6Sig scores. (C) The TCGA cohort confirmed that patients with higher m6Sig scores was associated with prolonged disease free survival (DFS).

## Supplementary Tables

| **Supplemental Table S1. Summary of Clinical characteristics of patients with melanoma in TCGA and Meta-cohort** | | | | | | |
| --- | --- | --- | --- | --- | --- | --- |
| **Dataset** | TCGA | GSE19234 | GSE22154 | GSE50509 | GSE59455 | GSE65904 |
| **Annotation** | TCGA-SKCM | Meta-Cohort | | | | |
| **No. of patients** | 394 | 38 | 22 | 19 | 141 | 214 |
| **Platform** | Illumina RNAseq | Affymetrix U133 Plus 2.0 Array | Illumina HumanHT-12 V3.0 | Illumina HumanHT-12 V4.0 | Illumina HumanRef-8 WG-DASL v3.0 | Illumina HumanHT-12 V4.0 |
| **Age (years)** |  |  |  |  |  |  |
| Range | 34-90 | 30-92 | 41-75 | 17-80 | 23-95 | 22-91 |
| Median | 68 | 63 | 58 | 59 | 68 | 64 |
| **Gender** |  |  |  |  |  |  |
| Female | 172 | 14 | 11 | 7 | 45 | 89 |
| Male | 281 | 24 | 11 | 12 | 96 | 124 |
| **Stage** |  |  |  |  |  |  |
| Metastatic | 348 | 5 | 22 | 19 | 102 | 188 |
| Primary | 103 | 33 | 0 | 0 | 39 | 16 |
| **m^6^A cluster** |  |  |  |  |  |  |
| C1 | 185 | 16 | 11 | 8 | 77 | 83 |
| C2 | 176 | 16 | 9 | 8 | 48 | 87 |
| C3 | 92 | 6 | 2 | 3 | 16 | 44 |
| **Overall survival (months)** |  |  |  |  |  |  |
| Range | 0-150 | 9.4-189.2 | 0.2-38.3 | 6.6-47.5 | 0.7-278.8 | 0.2-215.1 |
| Median | 19.3 | 38.1 | 4.1 | 18.8 | 32.1 | 17.8 |

| **Supplementary Table S2. Identification of significantly mutated genes in melanoma.** | | | | | | | | | | | | | |
| --- | --- | --- | --- | --- | --- | --- | --- | --- | --- | --- | --- | --- | --- |
| **gene** | **expr** | **reptime** | **hic** | **N_nonsilent** | **N_silent** | **N_noncoding** | **n_nonsilent** | **n_noncoding** | **nnei** | **x** | **X** | **p** | **q** |
| **BRAF** | 305191 | 616 | 25 | 2302420 | 649475 | 0 | 157 | 0 | 2 | 16 | 1613105 | 0 | 0 |
| **PPP6C** | 951081 | 168 | 46 | 1033350 | 282615 | 0 | 26 | 0 | 31 | 46 | 15042090 | 0 | 0 |
| **PTEN** | 259678 | 300 | 34 | 1256620 | 292160 | 0 | 25 | 0 | 50 | 75 | 21260450 | 0 | 0 |
| **TP53** | 2069567 | 213 | 34 | 1281935 | 353995 | 0 | 76 | 0 | 29 | 37 | 11942870 | 0 | 0 |
| **NRAS** | 600650 | 472 | 11 | 583905 | 150645 | 0 | 83 | 0 | 1 | 10 | 756130 | 1.55E-15 | 2.67E-12 |
| **CDKN2A** | 225405 | 357 | -15 | 989775 | 257715 | 0 | 22 | 0 | 11 | 15 | 3786045 | 1.12E-09 | 1.63E-06 |
| **NF1** | 462449 | 396 | 26 | 8652335 | 2312380 | 0 | 72 | 0 | 24 | 43 | 10609060 | 2.70E-08 | 3.18E-05 |
| **MAP2K1** | 506284 | 172 | 43 | 1199765 | 324115 | 0 | 19 | 0 | 17 | 21 | 6967435 | 2.66118E-05 | 0.01251829 |
| **RAPGEF5** | 747975 | 629 | 4 | 2293290 | 551535 | 0 | 28 | 0 | 11 | 10 | 4132985 | 3.23885E-05 | 0.024663455 |
| **B2M** | 673122 | 428 | 36 | 367690 | 97940 | 0 | 6 | 0 | 6 | 18 | 4117215 | 0.000240173 | 0.04923359 |
| **RCAN2** | 55610 | 819 | 8 | 585980 | 168490 | 0 | 8 | 0 | 38 | 123 | 23488585 | 0.000253565 | 0.05088019 |
| **ITGA4** | 233378 | 518 | 20 | 3174335 | 818380 | 0 | 34 | 0 | 9 | 16 | 4806530 | 0.000291397 | 0.05630985 |
| **SIRPB1** | 421731 | 424 | 39 | 2249715 | 685995 | 0 | 23 | 0 | 50 | 95 | 25652810 | 0.000306254 | 0.05719372 |
| **RB1** | 349930 | 450 | 41 | 2866820 | 732475 | 0 | 18 | 0 | 30 | 53 | 15472445 | 0.000316105 | 0.05786956 |
| **PPIAL4G** | 119504 | 375 | 34 | 489285 | 129480 | 0 | 5 | 0 | 50 | 71 | 23691520 | 0.000323618 | 0.05786956 |
| **RAC1** | 52413 | 215 | -22 | 641175 | 180525 | 0 | 9 | 0 | 50 | 261 | 46396585 | 0.000328281 | 0.05786956 |
| **KNSTRN** | 754243 | 197 | 47 | 554064 | 183792 | 0 | 19 | 0 | 50 | 58 | 17847984 | 0.00012409 | 0.03343692 |
| **COL9A2** | 627703 | 373 | 18 | 2053420 | 680600 | 0 | 13 | 0 | 25 | 37 | 12235445 | 0.000369904 | 0.06401043 |
| **CTNNB1** | 305811 | 448 | 8 | 2322340 | 673130 | 0 | 16 | 0 | 32 | 48 | 13650595 | 0.000403352 | 0.06792878 |
| **ZFX** | 615356 | NA | 50 | 2501205 | 617520 | 0 | 11 | 0 | 10 | 19 | 6230395 | 0.000446937 | 0.07267353 |
| **RQCD1** | 939398 | 179 | 48 | 891420 | 262695 | 0 | 7 | 0 | 22 | 33 | 8815430 | 0.000481233 | 0.07501664 |
| **MSR1** | 53956 | 947 | -48 | 1423450 | 366860 | 0 | 43 | 0 | 1 | 7 | 732060 | 0.000506654 | 0.07833209 |
| **GNAI2** | 846254 | 199 | 44 | 1103485 | 277220 | 0 | 9 | 0 | 11 | 19 | 4812755 | 0.00052952 | 0.08033296 |
| **ACD** | 1065785 | 147 | 41 | 1584470 | 527050 | 0 | 5 | 0 | 49 | 84 | 31164425 | 0.000550751 | 0.08173793 |
| **IDH1** | 260367 | 539 | 17 | 1271145 | 318720 | 0 | 8 | 0 | 11 | 18 | 4843880 | 0.000590609 | 0.08503864 |
| **TBC1D3B** | 739634 | 565 | 19 | 1655850 | 461895 | 0 | 4 | 0 | 3 | 1 | 1372405 | 0.000602885 | 0.08614865 |
| **OXA1L** | 1153069 | 351 | 36 | 1457480 | 439900 | 0 | 3 | 0 | 2 | 5 | 1652530 | 0.000637274 | 0.08903901 |
| **CCDC28A** | 402265 | 482 | 22 | 820455 | 231570 | 0 | 10 | 0 | 0 | 6 | 231570 | 0.000655656 | 0.09093375 |
| **FAM58A** | 940418 | NA | 24 | 733305 | 215385 | 0 | 4 | 0 | 50 | 68 | 20432110 | 0.000683523 | 0.09396094 |
| **STK19** | 2314693 | 203 | 34 | 1121330 | 355240 | 0 | 5 | 0 | 31 | 63 | 18711105 | 0.000692428 | 0.09396094 |

| **Supplementary Table S3. Prognostic analysis of 626 m6A-related DEGs using a univariate Cox analysis.** | | | | | | |
| --- | --- | --- | --- | --- | --- | --- |
| **Beta** | **HR** | **HR_95%CI Low** | **HR_95%CI High** | **HR (95% CI for HR)** | **p.value** | **Gene_Symbol** |
| 0.522 | 1.685 | 1.283 | 2.213 | 1.685 (1.283-2.213) | 0 | CFLAR |
| 0.638 | 1.893 | 1.44 | 2.489 | 1.893 (1.440-2.489) | 0 | CD38 |
| 0.641 | 1.898 | 1.443 | 2.497 | 1.898 (1.443-2.497) | 0 | ITGAL |
| 0.601 | 1.824 | 1.387 | 2.398 | 1.824 (1.387-2.398) | 0 | CEACAM21 |
| 0.519 | 1.68 | 1.277 | 2.211 | 1.680 (1.277-2.211) | 0 | IL32 |
| 0.636 | 1.889 | 1.436 | 2.485 | 1.889 (1.436-2.485) | 0 | TRAF3IP3 |
| 0.569 | 1.767 | 1.346 | 2.32 | 1.767 (1.346-2.320) | 0 | CD4 |
| 0.559 | 1.75 | 1.332 | 2.297 | 1.750 (1.332-2.297) | 0 | BTK |
| 0.495 | 1.641 | 1.249 | 2.158 | 1.641 (1.249-2.158) | 0 | ZBTB32 |
| 0.609 | 1.838 | 1.399 | 2.415 | 1.838 (1.399-2.415) | 0 | ALOX5 |
| 0.555 | 1.742 | 1.324 | 2.292 | 1.742 (1.324-2.292) | 0 | CD6 |
| 0.591 | 1.806 | 1.372 | 2.379 | 1.806 (1.372-2.379) | 0 | WAS |
| 0.666 | 1.946 | 1.476 | 2.566 | 1.946 (1.476-2.566) | 0 | CD74 |
| 0.707 | 2.027 | 1.543 | 2.663 | 2.027 (1.543-2.663) | 0 | BIRC3 |
| 0.559 | 1.749 | 1.33 | 2.301 | 1.749 (1.330-2.301) | 0 | NR1H3 |
| 0.496 | 1.642 | 1.251 | 2.155 | 1.642 (1.251-2.155) | 0 | TNFRSF1B |
| 0.551 | 1.734 | 1.321 | 2.277 | 1.734 (1.321-2.277) | 0 | POU2F2 |
| 0.633 | 1.883 | 1.431 | 2.477 | 1.883 (1.431-2.477) | 0 | STAP1 |
| 0.551 | 1.735 | 1.323 | 2.277 | 1.735 (1.323-2.277) | 0 | ADAM28 |
| 0.633 | 1.884 | 1.434 | 2.476 | 1.884 (1.434-2.476) | 0 | LCP2 |
| 0.731 | 2.078 | 1.578 | 2.736 | 2.078 (1.578-2.736) | 0 | TNFRSF9 |
| 0.641 | 1.897 | 1.442 | 2.496 | 1.897 (1.442-2.496) | 0 | LY75 |
| 0.527 | 1.694 | 1.288 | 2.227 | 1.694 (1.288-2.227) | 0 | TSPAN32 |
| 0.662 | 1.938 | 1.475 | 2.547 | 1.938 (1.475-2.547) | 0 | CD84 |
| 0.54 | 1.717 | 1.306 | 2.255 | 1.717 (1.306-2.255) | 0 | SPI1 |
| 0.577 | 1.781 | 1.355 | 2.34 | 1.781 (1.355-2.340) | 0 | DAPP1 |
| 0.518 | 1.679 | 1.274 | 2.212 | 1.679 (1.274-2.212) | 0 | ACAP1 |
| 0.557 | 1.746 | 1.326 | 2.298 | 1.746 (1.326-2.298) | 0 | TBX21 |
| 0.669 | 1.952 | 1.484 | 2.568 | 1.952 (1.484-2.568) | 0 | IPCEF1 |
| 0.508 | 1.662 | 1.268 | 2.179 | 1.662 (1.268-2.179) | 0 | ARHGAP15 |
| 0.693 | 2 | 1.519 | 2.632 | 2.000 (1.519-2.632) | 0 | APBB1IP |
| 0.675 | 1.963 | 1.493 | 2.582 | 1.963 (1.493-2.582) | 0 | P2RY10 |
| 0.598 | 1.818 | 1.382 | 2.392 | 1.818 (1.382-2.392) | 0 | SP140 |
| 0.69 | 1.994 | 1.517 | 2.621 | 1.994 (1.517-2.621) | 0 | PTPRC |
| 0.645 | 1.906 | 1.453 | 2.501 | 1.906 (1.453-2.501) | 0 | FYB |
| 0.638 | 1.893 | 1.439 | 2.489 | 1.893 (1.439-2.489) | 0 | CYLD |
| 0.527 | 1.694 | 1.291 | 2.222 | 1.694 (1.291-2.222) | 0 | PILRA |
| 0.586 | 1.796 | 1.366 | 2.361 | 1.796 (1.366-2.361) | 0 | LAT2 |
| 0.613 | 1.847 | 1.407 | 2.423 | 1.847 (1.407-2.423) | 0 | CASS4 |
| 0.582 | 1.79 | 1.362 | 2.354 | 1.790 (1.362-2.354) | 0 | SIGLEC1 |
| 0.656 | 1.927 | 1.462 | 2.54 | 1.927 (1.462-2.540) | 0 | SIRPG |
| 0.599 | 1.82 | 1.383 | 2.394 | 1.820 (1.383-2.394) | 0 | LAG3 |
| 0.581 | 1.787 | 1.362 | 2.345 | 1.787 (1.362-2.345) | 0 | LYZ |
| 0.651 | 1.918 | 1.46 | 2.52 | 1.918 (1.460-2.520) | 0 | NLRC4 |
| 0.533 | 1.704 | 1.299 | 2.234 | 1.704 (1.299-2.234) | 0 | SEL1L3 |
| 0.525 | 1.691 | 1.286 | 2.222 | 1.691 (1.286-2.222) | 0 | CECR1 |
| 0.618 | 1.854 | 1.409 | 2.441 | 1.854 (1.409-2.441) | 0 | BLNK |
| 0.554 | 1.741 | 1.323 | 2.291 | 1.741 (1.323-2.291) | 0 | IL12RB1 |
| 0.602 | 1.826 | 1.389 | 2.401 | 1.826 (1.389-2.401) | 0 | CYTH4 |
| 0.555 | 1.743 | 1.327 | 2.289 | 1.743 (1.327-2.289) | 0 | MFNG |
| 0.578 | 1.782 | 1.355 | 2.345 | 1.782 (1.355-2.345) | 0 | APOBEC3H |
| 0.567 | 1.763 | 1.339 | 2.32 | 1.763 (1.339-2.320) | 0 | NCF4 |
| 0.595 | 1.814 | 1.381 | 2.382 | 1.814 (1.381-2.382) | 0 | CSF2RB |
| 0.58 | 1.786 | 1.354 | 2.356 | 1.786 (1.354-2.356) | 0 | GZMH |
| 0.583 | 1.791 | 1.357 | 2.362 | 1.791 (1.357-2.362) | 0 | GZMB |
| 0.6 | 1.822 | 1.382 | 2.401 | 1.822 (1.382-2.401) | 0 | SLA2 |
| 0.542 | 1.719 | 1.309 | 2.258 | 1.719 (1.309-2.258) | 0 | HCK |
| 0.624 | 1.867 | 1.421 | 2.453 | 1.867 (1.421-2.453) | 0 | SAMHD1 |
| 0.687 | 1.988 | 1.512 | 2.614 | 1.988 (1.512-2.614) | 0 | TLR8 |
| 0.515 | 1.674 | 1.275 | 2.198 | 1.674 (1.275-2.198) | 0 | PIM2 |
| 0.497 | 1.644 | 1.252 | 2.157 | 1.644 (1.252-2.157) | 0 | CD40LG |
| 0.514 | 1.672 | 1.268 | 2.204 | 1.672 (1.268-2.204) | 0 | CORO1A |
| 0.759 | 2.136 | 1.623 | 2.812 | 2.136 (1.623-2.812) | 0 | IL21R |
| 0.5 | 1.648 | 1.254 | 2.167 | 1.648 (1.254-2.167) | 0 | RELB |
| 0.513 | 1.67 | 1.27 | 2.196 | 1.670 (1.270-2.196) | 0 | CD37 |
| 0.639 | 1.895 | 1.438 | 2.498 | 1.895 (1.438-2.498) | 0 | IL4I1 |
| 0.634 | 1.884 | 1.435 | 2.474 | 1.884 (1.435-2.474) | 0 | LILRB1 |
| 0.552 | 1.736 | 1.323 | 2.278 | 1.736 (1.323-2.278) | 0 | LILRA1 |
| 0.61 | 1.84 | 1.398 | 2.422 | 1.840 (1.398-2.422) | 0 | RASAL3 |
| 0.521 | 1.684 | 1.28 | 2.215 | 1.684 (1.280-2.215) | 0 | EBI3 |
| 0.727 | 2.068 | 1.568 | 2.728 | 2.068 (1.568-2.728) | 0 | DENND3 |
| 0.544 | 1.722 | 1.311 | 2.263 | 1.722 (1.311-2.263) | 0 | CD79A |
| 0.587 | 1.798 | 1.366 | 2.368 | 1.798 (1.366-2.368) | 0 | NKG7 |
| 0.623 | 1.865 | 1.418 | 2.453 | 1.865 (1.418-2.453) | 0 | CD33 |
| 0.568 | 1.764 | 1.344 | 2.315 | 1.764 (1.344-2.315) | 0 | PIK3CG |
| 0.713 | 2.04 | 1.553 | 2.68 | 2.040 (1.553-2.680) | 0 | TFEC |
| 0.582 | 1.79 | 1.362 | 2.351 | 1.790 (1.362-2.351) | 0 | GIMAP2 |
| 0.588 | 1.8 | 1.37 | 2.365 | 1.800 (1.370-2.365) | 0 | TMEM176B |
| 0.631 | 1.88 | 1.431 | 2.47 | 1.880 (1.431-2.470) | 0 | TNFSF8 |
| 0.514 | 1.672 | 1.274 | 2.194 | 1.672 (1.274-2.194) | 0 | DOCK8 |
| 0.706 | 2.026 | 1.535 | 2.672 | 2.026 (1.535-2.672) | 0 | RASSF4 |
| 0.564 | 1.757 | 1.338 | 2.309 | 1.757 (1.338-2.309) | 0 | C10orf54 |
| 0.698 | 2.009 | 1.525 | 2.646 | 2.009 (1.525-2.646) | 0 | SPOCK2 |
| 0.5 | 1.648 | 1.254 | 2.166 | 1.648 (1.254-2.166) | 0 | CCL2 |
| 0.621 | 1.86 | 1.415 | 2.446 | 1.860 (1.415-2.446) | 0 | ABI3 |
| 0.659 | 1.934 | 1.469 | 2.545 | 1.934 (1.469-2.545) | 0 | CLNK |
| 0.626 | 1.87 | 1.421 | 2.459 | 1.870 (1.421-2.459) | 0 | CRTAM |
| 0.717 | 2.048 | 1.556 | 2.695 | 2.048 (1.556-2.695) | 0 | MS4A6A |
| 0.556 | 1.743 | 1.328 | 2.288 | 1.743 (1.328-2.288) | 0 | IL10RA |
| 0.579 | 1.785 | 1.358 | 2.347 | 1.785 (1.358-2.347) | 0 | CD5 |
| 0.585 | 1.795 | 1.366 | 2.358 | 1.795 (1.366-2.358) | 0 | POU2AF1 |
| 0.655 | 1.926 | 1.464 | 2.533 | 1.926 (1.464-2.533) | 0 | CD69 |
| 0.549 | 1.732 | 1.319 | 2.275 | 1.732 (1.319-2.275) | 0 | SELPLG |
| 0.593 | 1.809 | 1.377 | 2.376 | 1.809 (1.377-2.376) | 0 | BIN2 |
| 0.624 | 1.866 | 1.417 | 2.456 | 1.866 (1.417-2.456) | 0 | PTPN6 |
| 0.522 | 1.686 | 1.285 | 2.213 | 1.686 (1.285-2.213) | 0 | CLEC4A |
| 0.552 | 1.737 | 1.324 | 2.28 | 1.737 (1.324-2.280) | 0 | KLRB1 |
| 0.619 | 1.856 | 1.412 | 2.441 | 1.856 (1.412-2.441) | 0 | SOD2 |
| 0.534 | 1.706 | 1.299 | 2.242 | 1.706 (1.299-2.242) | 0 | TREML2 |
| 0.634 | 1.885 | 1.433 | 2.478 | 1.885 (1.433-2.478) | 0 | VNN2 |
| 0.521 | 1.683 | 1.281 | 2.211 | 1.683 (1.281-2.211) | 0 | LY86 |
| 0.634 | 1.886 | 1.432 | 2.484 | 1.886 (1.432-2.484) | 0 | GZMK |
| 0.518 | 1.679 | 1.278 | 2.206 | 1.679 (1.278-2.206) | 0 | ITK |
| 0.558 | 1.747 | 1.332 | 2.292 | 1.747 (1.332-2.292) | 0 | ST8SIA4 |
| 0.71 | 2.035 | 1.547 | 2.677 | 2.035 (1.547-2.677) | 0 | CD86 |
| 0.557 | 1.746 | 1.329 | 2.294 | 1.746 (1.329-2.294) | 0 | CYTIP |
| 0.541 | 1.717 | 1.301 | 2.266 | 1.717 (1.301-2.266) | 0 | GNLY |
| 0.679 | 1.972 | 1.499 | 2.594 | 1.972 (1.499-2.594) | 0 | IL18RAP |
| 0.636 | 1.889 | 1.439 | 2.481 | 1.889 (1.439-2.481) | 0 | PLEK |
| 0.574 | 1.775 | 1.35 | 2.335 | 1.775 (1.350-2.335) | 0 | AMPD1 |
| 0.63 | 1.877 | 1.424 | 2.474 | 1.877 (1.424-2.474) | 0 | CD2 |
| 0.586 | 1.797 | 1.365 | 2.368 | 1.797 (1.365-2.368) | 0 | SLAMF1 |
| 0.549 | 1.731 | 1.317 | 2.275 | 1.731 (1.317-2.275) | 0 | CD48 |
| 0.715 | 2.044 | 1.554 | 2.69 | 2.044 (1.554-2.690) | 0 | PLA2G2D |
| 0.56 | 1.75 | 1.334 | 2.296 | 1.750 (1.334-2.296) | 0 | CR2 |
| 0.651 | 1.918 | 1.46 | 2.518 | 1.918 (1.460-2.518) | 0 | LRMP |
| 0.516 | 1.676 | 1.278 | 2.197 | 1.676 (1.278-2.197) | 0 | RARRES1 |
| 0.556 | 1.744 | 1.328 | 2.289 | 1.744 (1.328-2.289) | 0 | CSF3R |
| 0.747 | 2.111 | 1.602 | 2.781 | 2.111 (1.602-2.781) | 0 | CD274 |
| 0.571 | 1.769 | 1.349 | 2.321 | 1.769 (1.349-2.321) | 0 | CXorf21 |
| 0.524 | 1.688 | 1.283 | 2.222 | 1.688 (1.283-2.222) | 0 | PTK2B |
| 0.649 | 1.914 | 1.449 | 2.528 | 1.914 (1.449-2.528) | 0 | BCL2L14 |
| 0.79 | 2.204 | 1.674 | 2.902 | 2.204 (1.674-2.902) | 0 | CD80 |
| 0.592 | 1.807 | 1.376 | 2.372 | 1.807 (1.376-2.372) | 0 | CCR2 |
| 0.647 | 1.911 | 1.454 | 2.511 | 1.911 (1.454-2.511) | 0 | TNFSF10 |
| 0.618 | 1.855 | 1.41 | 2.439 | 1.855 (1.410-2.439) | 0 | TMEM156 |
| 0.515 | 1.674 | 1.273 | 2.201 | 1.674 (1.273-2.201) | 0 | FLT3 |
| 0.534 | 1.706 | 1.299 | 2.241 | 1.706 (1.299-2.241) | 0 | SASH3 |
| 0.66 | 1.934 | 1.472 | 2.541 | 1.934 (1.472-2.541) | 0 | LAX1 |
| 0.683 | 1.981 | 1.505 | 2.607 | 1.981 (1.505-2.607) | 0 | SRGN |
| 0.702 | 2.019 | 1.533 | 2.658 | 2.019 (1.533-2.658) | 0 | ARHGAP9 |
| 0.62 | 1.859 | 1.415 | 2.443 | 1.859 (1.415-2.443) | 0 | NCKAP1L |
| 0.669 | 1.952 | 1.483 | 2.568 | 1.952 (1.483-2.568) | 0 | ZNF831 |
| 0.574 | 1.776 | 1.348 | 2.34 | 1.776 (1.348-2.340) | 0 | ZBP1 |
| 0.766 | 2.15 | 1.626 | 2.842 | 2.150 (1.626-2.842) | 0 | IRF1 |
| 0.512 | 1.668 | 1.271 | 2.19 | 1.668 (1.271-2.190) | 0 | PTGER2 |
| 0.526 | 1.692 | 1.287 | 2.224 | 1.692 (1.287-2.224) | 0 | PSD4 |
| 0.5 | 1.648 | 1.253 | 2.168 | 1.648 (1.253-2.168) | 0 | HCST |
| 0.513 | 1.67 | 1.269 | 2.196 | 1.670 (1.269-2.196) | 0 | BCL11B |
| 0.629 | 1.875 | 1.426 | 2.467 | 1.875 (1.426-2.467) | 0 | APOL3 |
| 0.514 | 1.671 | 1.271 | 2.197 | 1.671 (1.271-2.197) | 0 | RAC2 |
| 0.549 | 1.732 | 1.316 | 2.278 | 1.732 (1.316-2.278) | 0 | TBC1D27 |
| 0.484 | 1.623 | 1.237 | 2.128 | 1.623 (1.237-2.128) | 0 | WDFY4 |
| 0.541 | 1.718 | 1.306 | 2.26 | 1.718 (1.306-2.260) | 0 | GMFG |
| 0.617 | 1.853 | 1.41 | 2.436 | 1.853 (1.410-2.436) | 0 | LILRB2 |
| 0.852 | 2.344 | 1.776 | 3.095 | 2.344 (1.776-3.095) | 0 | IDO1 |
| 0.576 | 1.779 | 1.352 | 2.341 | 1.779 (1.352-2.341) | 0 | NAPSB |
| 0.683 | 1.98 | 1.505 | 2.605 | 1.980 (1.505-2.605) | 0 | GCH1 |
| 0.701 | 2.016 | 1.531 | 2.654 | 2.016 (1.531-2.654) | 0 | TRIM22 |
| 0.563 | 1.756 | 1.336 | 2.307 | 1.756 (1.336-2.307) | 0 | PTPRE |
| 0.538 | 1.713 | 1.306 | 2.245 | 1.713 (1.306-2.245) | 0 | IGJ |
| 0.486 | 1.625 | 1.237 | 2.136 | 1.625 (1.237-2.136) | 0 | CLEC10A |
| 0.576 | 1.779 | 1.353 | 2.338 | 1.779 (1.353-2.338) | 0 | FCRL2 |
| 0.571 | 1.769 | 1.346 | 2.326 | 1.769 (1.346-2.326) | 0 | EPSTI1 |
| 0.568 | 1.765 | 1.346 | 2.316 | 1.765 (1.346-2.316) | 0 | GIMAP6 |
| 0.644 | 1.905 | 1.45 | 2.503 | 1.905 (1.450-2.503) | 0 | GIMAP4 |
| 0.548 | 1.729 | 1.316 | 2.272 | 1.729 (1.316-2.272) | 0 | ADAMDEC1 |
| 0.582 | 1.79 | 1.364 | 2.351 | 1.790 (1.364-2.351) | 0 | CD180 |
| 0.662 | 1.939 | 1.475 | 2.548 | 1.939 (1.475-2.548) | 0 | PTPN22 |
| 0.745 | 2.107 | 1.602 | 2.771 | 2.107 (1.602-2.771) | 0 | IL2RA |
| 0.621 | 1.862 | 1.416 | 2.448 | 1.862 (1.416-2.448) | 0 | IL15RA |
| 0.709 | 2.033 | 1.546 | 2.673 | 2.033 (1.546-2.673) | 0 | DOCK2 |
| 0.726 | 2.067 | 1.567 | 2.725 | 2.067 (1.567-2.725) | 0 | KLRD1 |
| 0.588 | 1.8 | 1.371 | 2.363 | 1.800 (1.371-2.363) | 0 | HAVCR2 |
| 0.535 | 1.708 | 1.301 | 2.243 | 1.708 (1.301-2.243) | 0 | LMO2 |
| 0.65 | 1.915 | 1.456 | 2.518 | 1.915 (1.456-2.518) | 0 | TESPA1 |
| 0.506 | 1.659 | 1.263 | 2.18 | 1.659 (1.263-2.180) | 0 | AGAP2 |
| 0.507 | 1.66 | 1.265 | 2.179 | 1.660 (1.265-2.179) | 0 | SP110 |
| 0.5 | 1.649 | 1.255 | 2.166 | 1.649 (1.255-2.166) | 0 | LCP1 |
| 0.595 | 1.813 | 1.381 | 2.381 | 1.813 (1.381-2.381) | 0 | AOAH |
| 0.704 | 2.021 | 1.532 | 2.668 | 2.021 (1.532-2.668) | 0 | SIT1 |
| 0.666 | 1.946 | 1.477 | 2.564 | 1.946 (1.477-2.564) | 0 | CD72 |
| 0.55 | 1.733 | 1.316 | 2.283 | 1.733 (1.316-2.283) | 0 | IL18BP |
| 0.713 | 2.041 | 1.55 | 2.687 | 2.041 (1.550-2.687) | 0 | STAT4 |
| 0.622 | 1.862 | 1.414 | 2.453 | 1.862 (1.414-2.453) | 0 | CXCL9 |
| 0.604 | 1.83 | 1.391 | 2.407 | 1.830 (1.391-2.407) | 0 | PARVG |
| 0.568 | 1.765 | 1.341 | 2.323 | 1.765 (1.341-2.323) | 0 | CD27 |
| 0.637 | 1.89 | 1.44 | 2.481 | 1.890 (1.440-2.481) | 0 | GPR65 |
| 0.616 | 1.852 | 1.409 | 2.434 | 1.852 (1.409-2.434) | 0 | IGSF6 |
| 0.627 | 1.872 | 1.419 | 2.469 | 1.872 (1.419-2.469) | 0 | NLRC5 |
| 0.655 | 1.926 | 1.464 | 2.533 | 1.926 (1.464-2.533) | 0 | IRF8 |
| 0.482 | 1.619 | 1.235 | 2.124 | 1.619 (1.235-2.124) | 0 | ARRB2 |
| 0.491 | 1.634 | 1.246 | 2.143 | 1.634 (1.246-2.143) | 0 | PIK3R5 |
| 0.579 | 1.784 | 1.358 | 2.342 | 1.784 (1.358-2.342) | 0 | VAV1 |
| 0.605 | 1.831 | 1.392 | 2.409 | 1.831 (1.392-2.409) | 0 | MYO1F |
| 0.748 | 2.113 | 1.603 | 2.783 | 2.113 (1.603-2.783) | 0 | SIGLEC10 |
| 0.544 | 1.722 | 1.311 | 2.262 | 1.722 (1.311-2.262) | 0 | C1orf162 |
| 0.607 | 1.835 | 1.396 | 2.412 | 1.835 (1.396-2.412) | 0 | CD53 |
| 0.498 | 1.646 | 1.254 | 2.16 | 1.646 (1.254-2.160) | 0 | GPA33 |
| 0.687 | 1.987 | 1.51 | 2.615 | 1.987 (1.510-2.615) | 0 | FCRL5 |
| 0.644 | 1.904 | 1.446 | 2.506 | 1.904 (1.446-2.506) | 0 | PTPN7 |
| 0.792 | 2.207 | 1.677 | 2.905 | 2.207 (1.677-2.905) | 0 | EAF2 |
| 0.541 | 1.717 | 1.309 | 2.253 | 1.717 (1.309-2.253) | 0 | 1-Mar |
| 0.623 | 1.864 | 1.414 | 2.457 | 1.864 (1.414-2.457) | 0 | GZMA |
| 0.726 | 2.067 | 1.571 | 2.719 | 2.067 (1.571-2.719) | 0 | TIMD4 |
| 0.566 | 1.761 | 1.34 | 2.316 | 1.761 (1.340-2.316) | 0 | FGD2 |
| 0.542 | 1.72 | 1.308 | 2.261 | 1.720 (1.308-2.261) | 0 | IL2RG |
| 0.545 | 1.724 | 1.312 | 2.266 | 1.724 (1.312-2.266) | 0 | DOK2 |
| 0.609 | 1.838 | 1.396 | 2.419 | 1.838 (1.396-2.419) | 0 | CD226 |
| 0.63 | 1.878 | 1.423 | 2.478 | 1.878 (1.423-2.478) | 0 | IL18 |
| 0.539 | 1.715 | 1.306 | 2.252 | 1.715 (1.306-2.252) | 0 | ARL11 |
| 0.501 | 1.651 | 1.259 | 2.165 | 1.651 (1.259-2.165) | 0 | PRDM8 |
| 0.611 | 1.843 | 1.404 | 2.42 | 1.843 (1.404-2.420) | 0 | BANK1 |
| 0.583 | 1.792 | 1.366 | 2.351 | 1.792 (1.366-2.351) | 0 | CD96 |
| 0.691 | 1.995 | 1.514 | 2.631 | 1.995 (1.514-2.631) | 0 | CD8A |
| 0.784 | 2.19 | 1.66 | 2.889 | 2.190 (1.660-2.889) | 0 | GBP5 |
| 0.569 | 1.767 | 1.346 | 2.318 | 1.767 (1.346-2.318) | 0 | PLCL2 |
| 0.607 | 1.835 | 1.397 | 2.41 | 1.835 (1.397-2.410) | 0 | SAMSN1 |
| 0.563 | 1.756 | 1.337 | 2.305 | 1.756 (1.337-2.305) | 0 | SLC7A7 |
| 0.504 | 1.655 | 1.262 | 2.171 | 1.655 (1.262-2.171) | 0 | PIK3AP1 |
| 0.555 | 1.741 | 1.326 | 2.286 | 1.741 (1.326-2.286) | 0 | SLA |
| 0.562 | 1.754 | 1.336 | 2.304 | 1.754 (1.336-2.304) | 0 | CXCL13 |
| 0.587 | 1.799 | 1.37 | 2.363 | 1.799 (1.370-2.363) | 0 | MS4A1 |
| 0.518 | 1.678 | 1.278 | 2.205 | 1.678 (1.278-2.205) | 0 | SUSD3 |
| 0.595 | 1.813 | 1.38 | 2.382 | 1.813 (1.380-2.382) | 0 | CD1D |
| 0.645 | 1.906 | 1.448 | 2.509 | 1.906 (1.448-2.509) | 0 | NCF1 |
| 0.636 | 1.889 | 1.434 | 2.488 | 1.889 (1.434-2.488) | 0 | SLAMF8 |
| 0.582 | 1.79 | 1.363 | 2.35 | 1.790 (1.363-2.350) | 0 | C1QC |
| 0.748 | 2.112 | 1.599 | 2.79 | 2.112 (1.599-2.790) | 0 | GPR114 |
| 0.57 | 1.769 | 1.342 | 2.332 | 1.769 (1.342-2.332) | 0 | RLTPR |
| 0.565 | 1.76 | 1.339 | 2.314 | 1.760 (1.339-2.314) | 0 | UBASH3A |
| 0.585 | 1.794 | 1.366 | 2.358 | 1.794 (1.366-2.358) | 0 | ITGB2 |
| 0.63 | 1.878 | 1.429 | 2.469 | 1.878 (1.429-2.469) | 0 | CD3G |
| 0.495 | 1.641 | 1.25 | 2.155 | 1.641 (1.250-2.155) | 0 | CXCR5 |
| 0.653 | 1.921 | 1.461 | 2.525 | 1.921 (1.461-2.525) | 0 | CCR5 |
| 0.681 | 1.977 | 1.501 | 2.603 | 1.977 (1.501-2.603) | 0 | FCRL3 |
| 0.499 | 1.647 | 1.255 | 2.161 | 1.647 (1.255-2.161) | 0 | HK3 |
| 0.673 | 1.959 | 1.491 | 2.575 | 1.959 (1.491-2.575) | 0 | IKZF3 |
| 0.602 | 1.826 | 1.39 | 2.399 | 1.826 (1.390-2.399) | 0 | SCIMP |
| 0.634 | 1.885 | 1.432 | 2.48 | 1.885 (1.432-2.480) | 0 | LAPTM5 |
| 0.528 | 1.695 | 1.292 | 2.225 | 1.695 (1.292-2.225) | 0 | VCAM1 |
| 0.605 | 1.831 | 1.392 | 2.409 | 1.831 (1.392-2.409) | 0 | SLAMF6 |
| 0.629 | 1.876 | 1.426 | 2.467 | 1.876 (1.426-2.467) | 0 | CTSS |
| 0.519 | 1.681 | 1.279 | 2.209 | 1.681 (1.279-2.209) | 0 | TNFAIP8L2 |
| 0.641 | 1.899 | 1.446 | 2.493 | 1.899 (1.446-2.493) | 0 | ARHGAP25 |
| 0.671 | 1.956 | 1.485 | 2.577 | 1.956 (1.485-2.577) | 0 | TRAT1 |
| 0.598 | 1.819 | 1.384 | 2.391 | 1.819 (1.384-2.391) | 0 | FCRL1 |
| 0.705 | 2.024 | 1.539 | 2.661 | 2.024 (1.539-2.661) | 0 | MNDA |
| 0.631 | 1.88 | 1.43 | 2.47 | 1.880 (1.430-2.470) | 0 | PYHIN1 |
| 0.607 | 1.835 | 1.399 | 2.408 | 1.835 (1.399-2.408) | 0 | CD200R1 |
| 0.623 | 1.864 | 1.417 | 2.451 | 1.864 (1.417-2.451) | 0 | SAMD3 |
| 0.669 | 1.952 | 1.486 | 2.566 | 1.952 (1.486-2.566) | 0 | TAGAP |
| 0.613 | 1.847 | 1.406 | 2.425 | 1.847 (1.406-2.425) | 0 | CYBB |
| 0.663 | 1.941 | 1.472 | 2.559 | 1.941 (1.472-2.559) | 0 | NCF1C |
| 0.606 | 1.833 | 1.394 | 2.41 | 1.833 (1.394-2.410) | 0 | GNGT2 |
| 0.618 | 1.856 | 1.412 | 2.439 | 1.856 (1.412-2.439) | 0 | SNX20 |
| 0.628 | 1.874 | 1.42 | 2.471 | 1.874 (1.420-2.471) | 0 | CD3D |
| 0.566 | 1.76 | 1.336 | 2.32 | 1.760 (1.336-2.320) | 0 | JSRP1 |
| 0.65 | 1.916 | 1.458 | 2.519 | 1.916 (1.458-2.519) | 0 | LAIR1 |
| 0.507 | 1.66 | 1.265 | 2.177 | 1.660 (1.265-2.177) | 0 | CD300A |
| 0.659 | 1.932 | 1.466 | 2.546 | 1.932 (1.466-2.546) | 0 | NLRC3 |
| 0.618 | 1.854 | 1.413 | 2.434 | 1.854 (1.413-2.434) | 0 | TRANK1 |
| 0.621 | 1.861 | 1.413 | 2.451 | 1.861 (1.413-2.451) | 0 | PNOC |
| 0.604 | 1.83 | 1.393 | 2.403 | 1.830 (1.393-2.403) | 0 | IRF2 |
| 0.677 | 1.967 | 1.496 | 2.586 | 1.967 (1.496-2.586) | 0 | MLKL |
| 0.672 | 1.957 | 1.489 | 2.573 | 1.957 (1.489-2.573) | 0 | CMAHP |
| 0.66 | 1.934 | 1.471 | 2.544 | 1.934 (1.471-2.544) | 0 | RHOH |
| 0.536 | 1.709 | 1.302 | 2.244 | 1.709 (1.302-2.244) | 0 | IL7R |
| 0.589 | 1.802 | 1.372 | 2.366 | 1.802 (1.372-2.366) | 0 | INPP5D |
| 0.593 | 1.809 | 1.378 | 2.375 | 1.809 (1.378-2.375) | 0 | RNASE6 |
| 0.577 | 1.78 | 1.355 | 2.34 | 1.780 (1.355-2.340) | 0 | MZB1 |
| 0.633 | 1.882 | 1.433 | 2.472 | 1.882 (1.433-2.472) | 0 | EMB |
| 0.553 | 1.738 | 1.325 | 2.279 | 1.738 (1.325-2.279) | 0 | PTGER4 |
| 0.524 | 1.689 | 1.286 | 2.218 | 1.689 (1.286-2.218) | 0 | P2RY6 |
| 0.666 | 1.947 | 1.478 | 2.565 | 1.947 (1.478-2.565) | 0 | CD8B |
| 0.543 | 1.72 | 1.309 | 2.261 | 1.720 (1.309-2.261) | 0 | CXCR6 |
| 0.648 | 1.911 | 1.454 | 2.512 | 1.911 (1.454-2.512) | 0 | CLEC7A |
| 0.511 | 1.667 | 1.266 | 2.194 | 1.667 (1.266-2.194) | 0 | CTSW |
| 0.533 | 1.704 | 1.298 | 2.238 | 1.704 (1.298-2.238) | 0 | RASGRP1 |
| 0.748 | 2.112 | 1.605 | 2.778 | 2.112 (1.605-2.778) | 0 | THEMIS |
| 0.513 | 1.67 | 1.272 | 2.193 | 1.670 (1.272-2.193) | 0 | RAB37 |
| 0.583 | 1.791 | 1.365 | 2.35 | 1.791 (1.365-2.350) | 0 | CYSLTR1 |
| 0.746 | 2.108 | 1.598 | 2.781 | 2.108 (1.598-2.781) | 0 | PARP15 |
| 0.589 | 1.803 | 1.374 | 2.366 | 1.803 (1.374-2.366) | 0 | ABCD2 |
| 0.56 | 1.75 | 1.333 | 2.297 | 1.750 (1.333-2.297) | 0 | C1QB |
| 0.525 | 1.691 | 1.289 | 2.219 | 1.691 (1.289-2.219) | 0 | C1QA |
| 0.531 | 1.701 | 1.294 | 2.235 | 1.701 (1.294-2.235) | 0 | XCR1 |
| 0.637 | 1.891 | 1.434 | 2.495 | 1.891 (1.434-2.495) | 0 | CD7 |
| 0.674 | 1.962 | 1.493 | 2.578 | 1.962 (1.493-2.578) | 0 | TLR10 |
| 0.548 | 1.73 | 1.319 | 2.269 | 1.730 (1.319-2.269) | 0 | P2RY14 |
| 0.703 | 2.019 | 1.531 | 2.662 | 2.019 (1.531-2.662) | 0 | GPR171 |
| 0.601 | 1.824 | 1.38 | 2.411 | 1.824 (1.380-2.411) | 0 | TBC1D10C |
| 0.541 | 1.717 | 1.309 | 2.253 | 1.717 (1.309-2.253) | 0 | KCNA3 |
| 0.501 | 1.651 | 1.257 | 2.168 | 1.651 (1.257-2.168) | 0 | CD19 |
| 0.485 | 1.625 | 1.237 | 2.135 | 1.625 (1.237-2.135) | 0 | ZC3H12D |
| 0.529 | 1.698 | 1.295 | 2.227 | 1.698 (1.295-2.227) | 0 | CD28 |
| 0.628 | 1.873 | 1.427 | 2.46 | 1.873 (1.427-2.460) | 0 | GIMAP7 |
| 0.67 | 1.954 | 1.483 | 2.575 | 1.954 (1.483-2.575) | 0 | HLA-DQB1 |
| 0.534 | 1.706 | 1.296 | 2.244 | 1.706 (1.296-2.244) | 0 | CIITA |
| 0.531 | 1.7 | 1.293 | 2.235 | 1.700 (1.293-2.235) | 0 | PCED1B |
| 0.668 | 1.951 | 1.485 | 2.561 | 1.951 (1.485-2.561) | 0 | AKAP5 |
| 0.53 | 1.7 | 1.296 | 2.229 | 1.700 (1.296-2.229) | 0 | TMEM150B |
| 0.617 | 1.853 | 1.409 | 2.438 | 1.853 (1.409-2.438) | 0 | HCLS1 |
| 0.59 | 1.804 | 1.369 | 2.378 | 1.804 (1.369-2.378) | 0 | C9orf139 |
| 0.578 | 1.782 | 1.355 | 2.344 | 1.782 (1.355-2.344) | 0 | PRF1 |
| 0.72 | 2.054 | 1.556 | 2.71 | 2.054 (1.556-2.710) | 0 | FCRL6 |
| 0.594 | 1.81 | 1.379 | 2.376 | 1.810 (1.379-2.376) | 0 | P2RY13 |
| 0.507 | 1.661 | 1.265 | 2.18 | 1.661 (1.265-2.180) | 0 | SLC9A9 |
| 0.606 | 1.832 | 1.393 | 2.41 | 1.832 (1.393-2.410) | 0 | TIGIT |
| 0.49 | 1.633 | 1.244 | 2.144 | 1.633 (1.244-2.144) | 0 | FAM159A |
| 0.654 | 1.924 | 1.462 | 2.53 | 1.924 (1.462-2.530) | 0 | NCF1B |
| 0.598 | 1.818 | 1.384 | 2.387 | 1.818 (1.384-2.387) | 0 | CSF1R |
| 0.611 | 1.842 | 1.397 | 2.428 | 1.842 (1.397-2.428) | 0 | LCK |
| 0.595 | 1.812 | 1.382 | 2.377 | 1.812 (1.382-2.377) | 0 | SLC8A1 |
| 0.686 | 1.987 | 1.513 | 2.609 | 1.987 (1.513-2.609) | 0 | FAM46C |
| 0.557 | 1.746 | 1.331 | 2.291 | 1.746 (1.331-2.291) | 0 | CCR4 |
| 0.756 | 2.13 | 1.616 | 2.808 | 2.130 (1.616-2.808) | 0 | SH2D1A |
| 0.73 | 2.075 | 1.577 | 2.729 | 2.075 (1.577-2.729) | 0 | CLECL1 |
| 0.62 | 1.858 | 1.408 | 2.453 | 1.858 (1.408-2.453) | 0 | STAC3 |
| 0.651 | 1.918 | 1.459 | 2.521 | 1.918 (1.459-2.521) | 0 | IKZF1 |
| 0.653 | 1.921 | 1.461 | 2.524 | 1.921 (1.461-2.524) | 0 | EVI2B |
| 0.574 | 1.775 | 1.35 | 2.333 | 1.775 (1.350-2.333) | 0 | C16orf54 |
| 0.565 | 1.76 | 1.339 | 2.313 | 1.760 (1.339-2.313) | 0 | CD300LF |
| 0.575 | 1.776 | 1.351 | 2.335 | 1.776 (1.351-2.335) | 0 | BTLA |
| 0.636 | 1.89 | 1.437 | 2.485 | 1.890 (1.437-2.485) | 0 | ARHGAP30 |
| 0.577 | 1.781 | 1.352 | 2.345 | 1.781 (1.352-2.345) | 0 | CXCR3 |
| 0.61 | 1.841 | 1.402 | 2.418 | 1.841 (1.402-2.418) | 0 | LILRB4 |
| 0.689 | 1.991 | 1.512 | 2.623 | 1.991 (1.512-2.623) | 0 | GCNT1 |
| 0.671 | 1.955 | 1.484 | 2.576 | 1.955 (1.484-2.576) | 0 | SEMA4D |
| 0.699 | 2.011 | 1.522 | 2.657 | 2.011 (1.522-2.657) | 0 | TTC24 |
| 0.6 | 1.822 | 1.387 | 2.395 | 1.822 (1.387-2.395) | 0 | CLEC17A |
| 0.584 | 1.794 | 1.362 | 2.362 | 1.794 (1.362-2.362) | 0 | PDCD1 |
| 0.522 | 1.686 | 1.285 | 2.212 | 1.686 (1.285-2.212) | 0 | SELL |
| 0.709 | 2.032 | 1.545 | 2.673 | 2.032 (1.545-2.673) | 0 | FAM26F |
| 0.669 | 1.952 | 1.486 | 2.565 | 1.952 (1.486-2.565) | 0 | BEND4 |
| 0.541 | 1.718 | 1.309 | 2.256 | 1.718 (1.309-2.256) | 0 | NUGGC |
| 0.713 | 2.04 | 1.547 | 2.689 | 2.040 (1.547-2.689) | 0 | HLA-DRB1 |
| 0.54 | 1.716 | 1.308 | 2.25 | 1.716 (1.308-2.250) | 0 | SIRPB2 |
| 0.668 | 1.95 | 1.484 | 2.563 | 1.950 (1.484-2.563) | 0 | GIMAP5 |
| 0.558 | 1.748 | 1.332 | 2.294 | 1.748 (1.332-2.294) | 0 | TLR7 |
| 0.55 | 1.733 | 1.318 | 2.278 | 1.733 (1.318-2.278) | 0 | HSH2D |
| 0.65 | 1.916 | 1.455 | 2.523 | 1.916 (1.455-2.523) | 0 | HLA-DQA1 |
| 0.657 | 1.929 | 1.468 | 2.535 | 1.929 (1.468-2.535) | 0 | DTHD1 |
| 0.547 | 1.729 | 1.315 | 2.271 | 1.729 (1.315-2.271) | 0 | SPN |
| 0.588 | 1.801 | 1.367 | 2.372 | 1.801 (1.367-2.372) | 0 | C5orf56 |
| 0.685 | 1.983 | 1.508 | 2.607 | 1.983 (1.508-2.607) | 0 | PDCD1LG2 |
| 0.522 | 1.686 | 1.282 | 2.217 | 1.686 (1.282-2.217) | 0 | CARD11 |
| 0.639 | 1.895 | 1.443 | 2.49 | 1.895 (1.443-2.490) | 0 | RCSD1 |
| 0.524 | 1.689 | 1.286 | 2.22 | 1.689 (1.286-2.220) | 0 | GRIN3A |
| 0.572 | 1.772 | 1.347 | 2.329 | 1.772 (1.347-2.329) | 0 | CD247 |
| 0.52 | 1.682 | 1.279 | 2.212 | 1.682 (1.279-2.212) | 0 | TOX |
| 0.589 | 1.802 | 1.368 | 2.375 | 1.802 (1.368-2.375) | 0 | CD3E |
| 0.492 | 1.636 | 1.247 | 2.146 | 1.636 (1.247-2.146) | 0 | C10orf128 |
| 0.729 | 2.074 | 1.574 | 2.733 | 2.074 (1.574-2.733) | 0 | HLA-DOA |
| 0.529 | 1.697 | 1.291 | 2.232 | 1.697 (1.291-2.232) | 0 | HLA-DMA |
| 0.666 | 1.947 | 1.477 | 2.566 | 1.947 (1.477-2.566) | 0 | HLA-DRA |
| 0.637 | 1.891 | 1.438 | 2.488 | 1.891 (1.438-2.488) | 0 | AIF1 |
| 0.54 | 1.715 | 1.307 | 2.251 | 1.715 (1.307-2.251) | 0 | NCR3 |
| 0.68 | 1.973 | 1.494 | 2.607 | 1.973 (1.494-2.607) | 0 | LST1 |
| 0.623 | 1.865 | 1.419 | 2.45 | 1.865 (1.419-2.450) | 0 | SLFN12L |
| 0.532 | 1.702 | 1.295 | 2.235 | 1.702 (1.295-2.235) | 0 | TMSB4X |
| 0.557 | 1.745 | 1.328 | 2.292 | 1.745 (1.328-2.292) | 0 | IGKC |
| 0.551 | 1.735 | 1.321 | 2.281 | 1.735 (1.321-2.281) | 0 | IGLC2 |
| 0.64 | 1.896 | 1.438 | 2.5 | 1.896 (1.438-2.500) | 0 | TRBV28 |
| 0.589 | 1.803 | 1.368 | 2.377 | 1.803 (1.368-2.377) | 0 | TRBC2 |
| 0.593 | 1.809 | 1.376 | 2.378 | 1.809 (1.376-2.378) | 0 | IGHG1 |
| 0.5 | 1.648 | 1.255 | 2.165 | 1.648 (1.255-2.165) | 0 | IGHG3 |
| 0.526 | 1.692 | 1.289 | 2.221 | 1.692 (1.289-2.221) | 0 | IGHM |
| 0.492 | 1.636 | 1.247 | 2.145 | 1.636 (1.247-2.145) | 0 | IGHV4-34 |
| 0.608 | 1.837 | 1.399 | 2.413 | 1.837 (1.399-2.413) | 0 | GIMAP1 |
| 0.661 | 1.937 | 1.467 | 2.557 | 1.937 (1.467-2.557) | 0 | HLA-DPB1 |
| 0.497 | 1.643 | 1.251 | 2.159 | 1.643 (1.251-2.159) | 0 | MIAT |
| 0.637 | 1.89 | 1.434 | 2.49 | 1.890 (1.434-2.490) | 0 | KIAA0125 |
| 0.598 | 1.819 | 1.382 | 2.394 | 1.819 (1.382-2.394) | 0 | LTA |
| 0.532 | 1.703 | 1.29 | 2.247 | 1.703 (1.290-2.247) | 0 | ITGB2-AS1 |
| 0.592 | 1.807 | 1.372 | 2.379 | 1.807 (1.372-2.379) | 0 | TRGC2 |
| 0.532 | 1.703 | 1.296 | 2.237 | 1.703 (1.296-2.237) | 0 | LTB |
| 0.556 | 1.744 | 1.327 | 2.291 | 1.744 (1.327-2.291) | 0 | RP5-1091N2.9 |
| 0.627 | 1.872 | 1.417 | 2.472 | 1.872 (1.417-2.472) | 0 | PATL2 |
| 0.711 | 2.036 | 1.547 | 2.679 | 2.036 (1.547-2.679) | 0 | CXCR2P1 |
| 0.725 | 2.064 | 1.564 | 2.724 | 2.064 (1.564-2.724) | 0 | HLA-DPA1 |
| 0.509 | 1.664 | 1.267 | 2.183 | 1.664 (1.267-2.183) | 0 | NFAM1 |
| 0.635 | 1.888 | 1.437 | 2.479 | 1.888 (1.437-2.479) | 0 | BHLHE40-AS1 |
| 0.576 | 1.779 | 1.352 | 2.34 | 1.779 (1.352-2.340) | 0 | AP003774.1 |
| 0.859 | 2.36 | 1.786 | 3.118 | 2.360 (1.786-3.118) | 0 | APOBEC3G |
| 0.514 | 1.672 | 1.272 | 2.197 | 1.672 (1.272-2.197) | 0 | TNFRSF13B |
| 0.565 | 1.76 | 1.336 | 2.318 | 1.760 (1.336-2.318) | 0 | HLA-DOB |
| 0.512 | 1.668 | 1.271 | 2.189 | 1.668 (1.271-2.189) | 0 | IGKV3-11 |
| 0.659 | 1.933 | 1.469 | 2.544 | 1.933 (1.469-2.544) | 0 | HLA-DMB |
| 0.516 | 1.676 | 1.277 | 2.199 | 1.676 (1.277-2.199) | 0 | IGKV1-5 |
| 0.661 | 1.937 | 1.471 | 2.549 | 1.937 (1.471-2.549) | 0 | APOBEC3D |
| 0.549 | 1.732 | 1.315 | 2.28 | 1.732 (1.315-2.280) | 0 | RP11-539L10.2 |
| 0.574 | 1.776 | 1.351 | 2.335 | 1.776 (1.351-2.335) | 0 | PCED1B-AS1 |
| 0.648 | 1.912 | 1.454 | 2.514 | 1.912 (1.454-2.514) | 0 | RP11-290F5.1 |
| 0.612 | 1.844 | 1.401 | 2.425 | 1.844 (1.401-2.425) | 0 | RP11-81H14.2 |
| 0.675 | 1.964 | 1.495 | 2.58 | 1.964 (1.495-2.580) | 0 | GVINP1 |
| 0.574 | 1.776 | 1.348 | 2.339 | 1.776 (1.348-2.339) | 0 | RP11-428G5.5 |
| 0.525 | 1.691 | 1.284 | 2.226 | 1.691 (1.284-2.226) | 0 | CTD-2547L24.3 |
| 0.523 | 1.688 | 1.281 | 2.223 | 1.688 (1.281-2.223) | 0 | CTC-378H22.2 |
| 0.688 | 1.991 | 1.516 | 2.613 | 1.991 (1.516-2.613) | 0 | RP11-284N8.3 |
| 0.589 | 1.803 | 1.371 | 2.37 | 1.803 (1.371-2.370) | 0 | AC009133.17 |
| 0.528 | 1.695 | 1.29 | 2.229 | 1.695 (1.290-2.229) | 0 | C1orf186 |
| 0.531 | 1.701 | 1.295 | 2.234 | 1.701 (1.295-2.234) | 0 | RASSF5 |
| 0.714 | 2.041 | 1.549 | 2.69 | 2.041 (1.549-2.690) | 0 | RP11-1094M14.8 |
| 0.693 | 2.001 | 1.519 | 2.636 | 2.001 (1.519-2.636) | 0 | AC006129.2 |
| 0.567 | 1.762 | 1.339 | 2.32 | 1.762 (1.339-2.320) | 0 | CCL5 |
| 0.665 | 1.944 | 1.476 | 2.561 | 1.944 (1.476-2.561) | 0 | CCL4 |
| 0.539 | 1.714 | 1.302 | 2.258 | 1.714 (1.302-2.258) | 0 | PIK3R6 |
| 0.633 | 1.883 | 1.428 | 2.482 | 1.883 (1.428-2.482) | 0 | TRAC |
| 0.493 | 1.638 | 1.249 | 2.148 | 1.638 (1.249-2.148) | 0 | SSTR3 |
| 0.568 | 1.764 | 1.337 | 2.328 | 1.764 (1.337-2.328) | 0 | AD000864.6 |
| 0.503 | 1.653 | 1.257 | 2.174 | 1.653 (1.257-2.174) | 0 | AC133644.2 |
| 0.583 | 1.792 | 1.365 | 2.353 | 1.792 (1.365-2.353) | 0 | TRG-AS1 |
| 0.467 | 1.595 | 1.214 | 2.095 | 1.595 (1.214-2.095) | 0.001 | FGR |
| 0.475 | 1.607 | 1.225 | 2.109 | 1.607 (1.225-2.109) | 0.001 | TMEM176A |
| 0.468 | 1.596 | 1.215 | 2.098 | 1.596 (1.215-2.098) | 0.001 | SLAMF7 |
| 0.46 | 1.583 | 1.206 | 2.079 | 1.583 (1.206-2.079) | 0.001 | ICAM3 |
| 0.464 | 1.59 | 1.21 | 2.091 | 1.590 (1.210-2.091) | 0.001 | CST7 |
| 0.451 | 1.57 | 1.195 | 2.062 | 1.570 (1.195-2.062) | 0.001 | LAMP3 |
| 0.457 | 1.579 | 1.203 | 2.072 | 1.579 (1.203-2.072) | 0.001 | ITM2A |
| 0.466 | 1.594 | 1.214 | 2.092 | 1.594 (1.214-2.092) | 0.001 | IL2RB |
| 0.453 | 1.573 | 1.199 | 2.064 | 1.573 (1.199-2.064) | 0.001 | SUSD6 |
| 0.46 | 1.584 | 1.207 | 2.078 | 1.584 (1.207-2.078) | 0.001 | CD40 |
| 0.443 | 1.557 | 1.187 | 2.043 | 1.557 (1.187-2.043) | 0.001 | FCER2 |
| 0.468 | 1.596 | 1.218 | 2.092 | 1.596 (1.218-2.092) | 0.001 | MS4A4A |
| 0.457 | 1.579 | 1.203 | 2.073 | 1.579 (1.203-2.073) | 0.001 | C11orf21 |
| 0.454 | 1.575 | 1.202 | 2.064 | 1.575 (1.202-2.064) | 0.001 | MAN1A1 |
| 0.446 | 1.562 | 1.19 | 2.05 | 1.562 (1.190-2.050) | 0.001 | CCR6 |
| 0.46 | 1.584 | 1.209 | 2.076 | 1.584 (1.209-2.076) | 0.001 | IL18R1 |
| 0.463 | 1.589 | 1.211 | 2.085 | 1.589 (1.211-2.085) | 0.001 | KMO |
| 0.464 | 1.591 | 1.212 | 2.089 | 1.591 (1.212-2.089) | 0.001 | CCND2 |
| 0.469 | 1.598 | 1.219 | 2.095 | 1.598 (1.219-2.095) | 0.001 | CCRL2 |
| 0.47 | 1.601 | 1.219 | 2.102 | 1.601 (1.219-2.102) | 0.001 | TOX2 |
| 0.474 | 1.606 | 1.222 | 2.111 | 1.606 (1.222-2.111) | 0.001 | GPR18 |
| 0.451 | 1.57 | 1.195 | 2.063 | 1.570 (1.195-2.063) | 0.001 | FGD3 |
| 0.473 | 1.605 | 1.224 | 2.105 | 1.605 (1.224-2.105) | 0.001 | SIGLEC9 |
| 0.451 | 1.57 | 1.198 | 2.057 | 1.570 (1.198-2.057) | 0.001 | SLCO2B1 |
| 0.446 | 1.562 | 1.187 | 2.056 | 1.562 (1.187-2.056) | 0.001 | PSTPIP1 |
| 0.447 | 1.564 | 1.193 | 2.05 | 1.564 (1.193-2.050) | 0.001 | SKAP1 |
| 0.465 | 1.592 | 1.214 | 2.088 | 1.592 (1.214-2.088) | 0.001 | TNFAIP8 |
| 0.477 | 1.611 | 1.228 | 2.113 | 1.611 (1.228-2.113) | 0.001 | GAB3 |
| 0.48 | 1.616 | 1.232 | 2.121 | 1.616 (1.232-2.121) | 0.001 | GFI1 |
| 0.468 | 1.596 | 1.216 | 2.094 | 1.596 (1.216-2.094) | 0.001 | ICOS |
| 0.462 | 1.587 | 1.211 | 2.081 | 1.587 (1.211-2.081) | 0.001 | ARHGEF3 |
| 0.466 | 1.594 | 1.215 | 2.091 | 1.594 (1.215-2.091) | 0.001 | TSHR |
| 0.475 | 1.608 | 1.225 | 2.111 | 1.608 (1.225-2.111) | 0.001 | SMCO4 |
| 0.474 | 1.607 | 1.219 | 2.119 | 1.607 (1.219-2.119) | 0.001 | TVP23A |
| 0.467 | 1.595 | 1.215 | 2.094 | 1.595 (1.215-2.094) | 0.001 | FAM129C |
| 0.471 | 1.602 | 1.218 | 2.107 | 1.602 (1.218-2.107) | 0.001 | TMC8 |
| 0.482 | 1.619 | 1.23 | 2.13 | 1.619 (1.230-2.130) | 0.001 | CCDC88B |
| 0.452 | 1.571 | 1.197 | 2.062 | 1.571 (1.197-2.062) | 0.001 | LGALS9 |
| 0.468 | 1.597 | 1.218 | 2.094 | 1.597 (1.218-2.094) | 0.001 | SIGLEC7 |
| 0.461 | 1.585 | 1.208 | 2.08 | 1.585 (1.208-2.080) | 0.001 | PTAFR |
| 0.462 | 1.587 | 1.208 | 2.084 | 1.587 (1.208-2.084) | 0.001 | CD52 |
| 0.47 | 1.6 | 1.218 | 2.101 | 1.600 (1.218-2.101) | 0.001 | KLHL6 |
| 0.444 | 1.558 | 1.187 | 2.046 | 1.558 (1.187-2.046) | 0.001 | GLRX |
| 0.442 | 1.555 | 1.186 | 2.039 | 1.555 (1.186-2.039) | 0.001 | CMKLR1 |
| 0.481 | 1.618 | 1.233 | 2.124 | 1.618 (1.233-2.124) | 0.001 | LRRC25 |
| 0.476 | 1.61 | 1.224 | 2.119 | 1.610 (1.224-2.119) | 0.001 | 1-Sep |
| 0.483 | 1.621 | 1.235 | 2.127 | 1.621 (1.235-2.127) | 0.001 | P2RY8 |
| 0.486 | 1.625 | 1.236 | 2.137 | 1.625 (1.236-2.137) | 0.001 | LPAR5 |
| 0.466 | 1.594 | 1.213 | 2.094 | 1.594 (1.213-2.094) | 0.001 | PRR33 |
| 0.482 | 1.62 | 1.233 | 2.127 | 1.620 (1.233-2.127) | 0.001 | FAM179A |
| 0.481 | 1.617 | 1.233 | 2.121 | 1.617 (1.233-2.121) | 0.001 | MPEG1 |
| 0.447 | 1.563 | 1.193 | 2.048 | 1.563 (1.193-2.048) | 0.001 | CR1 |
| 0.46 | 1.584 | 1.208 | 2.078 | 1.584 (1.208-2.078) | 0.001 | GGTA1P |
| 0.467 | 1.596 | 1.213 | 2.1 | 1.596 (1.213-2.100) | 0.001 | EXOC3L4 |
| 0.482 | 1.619 | 1.231 | 2.128 | 1.619 (1.231-2.128) | 0.001 | DENND1C |
| 0.454 | 1.574 | 1.2 | 2.065 | 1.574 (1.200-2.065) | 0.001 | IGLV1-51 |
| 0.461 | 1.586 | 1.206 | 2.086 | 1.586 (1.206-2.086) | 0.001 | TRDC |
| 0.48 | 1.615 | 1.23 | 2.121 | 1.615 (1.230-2.121) | 0.001 | AC079767.4 |
| 0.476 | 1.61 | 1.225 | 2.116 | 1.610 (1.225-2.116) | 0.001 | RP11-693N9.2 |
| 0.472 | 1.603 | 1.224 | 2.101 | 1.603 (1.224-2.101) | 0.001 | IFNG-AS1 |
| 0.428 | 1.534 | 1.168 | 2.016 | 1.534 (1.168-2.016) | 0.002 | RNASET2 |
| 0.427 | 1.533 | 1.169 | 2.01 | 1.533 (1.169-2.010) | 0.002 | PRKCQ |
| 0.419 | 1.521 | 1.16 | 1.994 | 1.521 (1.160-1.994) | 0.002 | NCF2 |
| 0.425 | 1.53 | 1.166 | 2.008 | 1.530 (1.166-2.008) | 0.002 | CD244 |
| 0.434 | 1.543 | 1.177 | 2.022 | 1.543 (1.177-2.022) | 0.002 | HS3ST3B1 |
| 0.431 | 1.539 | 1.17 | 2.025 | 1.539 (1.170-2.025) | 0.002 | S1PR4 |
| 0.437 | 1.548 | 1.181 | 2.027 | 1.548 (1.181-2.027) | 0.002 | AOX1 |
| 0.441 | 1.554 | 1.183 | 2.041 | 1.554 (1.183-2.041) | 0.002 | NAAA |
| 0.434 | 1.543 | 1.175 | 2.027 | 1.543 (1.175-2.027) | 0.002 | AMICA1 |
| 0.433 | 1.543 | 1.177 | 2.021 | 1.543 (1.177-2.021) | 0.002 | GIMAP8 |
| 0.435 | 1.545 | 1.178 | 2.026 | 1.545 (1.178-2.026) | 0.002 | RNF213 |
| 0.438 | 1.549 | 1.18 | 2.033 | 1.549 (1.180-2.033) | 0.002 | UCP2 |
| 0.434 | 1.543 | 1.177 | 2.024 | 1.543 (1.177-2.024) | 0.002 | GAPT |
| 0.447 | 1.564 | 1.185 | 2.063 | 1.564 (1.185-2.063) | 0.002 | SOCS1 |
| 0.426 | 1.532 | 1.167 | 2.01 | 1.532 (1.167-2.010) | 0.002 | CNR2 |
| 0.431 | 1.54 | 1.172 | 2.022 | 1.540 (1.172-2.022) | 0.002 | IGHA1 |
| 0.423 | 1.527 | 1.163 | 2.003 | 1.527 (1.163-2.003) | 0.002 | IGKV3D-15 |
| 0.422 | 1.526 | 1.163 | 2.002 | 1.526 (1.163-2.002) | 0.002 | PRKCQ-AS1 |
| 0.438 | 1.55 | 1.181 | 2.036 | 1.550 (1.181-2.036) | 0.002 | RP11-18H21.1 |
| 0.413 | 1.511 | 1.151 | 1.983 | 1.511 (1.151-1.983) | 0.003 | FOXP3 |
| 0.415 | 1.514 | 1.155 | 1.984 | 1.514 (1.155-1.984) | 0.003 | PRDM1 |
| 0.407 | 1.502 | 1.145 | 1.97 | 1.502 (1.145-1.970) | 0.003 | P2RX5 |
| 0.417 | 1.517 | 1.158 | 1.987 | 1.517 (1.158-1.987) | 0.003 | ACRBP |
| 0.409 | 1.505 | 1.144 | 1.98 | 1.505 (1.144-1.980) | 0.003 | ZAP70 |
| 0.417 | 1.518 | 1.157 | 1.993 | 1.518 (1.157-1.993) | 0.003 | LY9 |
| 0.41 | 1.507 | 1.149 | 1.976 | 1.507 (1.149-1.976) | 0.003 | PACSIN1 |
| 0.411 | 1.509 | 1.15 | 1.979 | 1.509 (1.150-1.979) | 0.003 | LSP1 |
| 0.418 | 1.518 | 1.154 | 1.998 | 1.518 (1.154-1.998) | 0.003 | BLK |
| 0.414 | 1.514 | 1.155 | 1.984 | 1.514 (1.155-1.984) | 0.003 | SLC2A5 |
| 0.406 | 1.501 | 1.145 | 1.968 | 1.501 (1.145-1.968) | 0.003 | PLAC8 |
| 0.416 | 1.516 | 1.154 | 1.991 | 1.516 (1.154-1.991) | 0.003 | SYTL3 |
| 0.407 | 1.503 | 1.144 | 1.974 | 1.503 (1.144-1.974) | 0.003 | PLD4 |
| 0.412 | 1.509 | 1.148 | 1.983 | 1.509 (1.148-1.983) | 0.003 | MEI1 |
| 0.409 | 1.505 | 1.149 | 1.972 | 1.505 (1.149-1.972) | 0.003 | GPR183 |
| 0.405 | 1.5 | 1.143 | 1.967 | 1.500 (1.143-1.967) | 0.003 | C19orf38 |
| 0.415 | 1.515 | 1.156 | 1.986 | 1.515 (1.156-1.986) | 0.003 | LYN |
| 0.413 | 1.512 | 1.151 | 1.986 | 1.512 (1.151-1.986) | 0.003 | IGLL5 |
| 0.416 | 1.516 | 1.149 | 2 | 1.516 (1.149-2.000) | 0.003 | RP11-876N24.3 |
| 0.396 | 1.486 | 1.133 | 1.948 | 1.486 (1.133-1.948) | 0.004 | COL4A4 |
| 0.407 | 1.502 | 1.143 | 1.975 | 1.502 (1.143-1.975) | 0.004 | FLT3LG |
| 0.401 | 1.493 | 1.134 | 1.965 | 1.493 (1.134-1.965) | 0.004 | ABCG1 |
| 0.401 | 1.493 | 1.139 | 1.957 | 1.493 (1.139-1.957) | 0.004 | NLRP3 |
| 0.392 | 1.48 | 1.13 | 1.939 | 1.480 (1.130-1.939) | 0.004 | ACSL5 |
| 0.396 | 1.487 | 1.132 | 1.952 | 1.487 (1.132-1.952) | 0.004 | IGHG2 |
| 0.403 | 1.496 | 1.139 | 1.966 | 1.496 (1.139-1.966) | 0.004 | RP11-47L3.1 |
| 0.388 | 1.473 | 1.125 | 1.93 | 1.473 (1.125-1.930) | 0.005 | FAM107B |
| 0.392 | 1.48 | 1.127 | 1.942 | 1.480 (1.127-1.942) | 0.005 | JAK3 |
| 0.391 | 1.478 | 1.128 | 1.937 | 1.478 (1.128-1.937) | 0.005 | PRR5L |
| 0.396 | 1.486 | 1.13 | 1.953 | 1.486 (1.130-1.953) | 0.005 | SEMA4A |
| 0.382 | 1.465 | 1.113 | 1.928 | 1.465 (1.113-1.928) | 0.006 | KIAA1324 |
| 0.378 | 1.459 | 1.113 | 1.913 | 1.459 (1.113-1.913) | 0.006 | ITGAX |
| 0.379 | 1.461 | 1.115 | 1.915 | 1.461 (1.115-1.915) | 0.006 | FLI1 |
| 0.378 | 1.459 | 1.113 | 1.913 | 1.459 (1.113-1.913) | 0.006 | PAX5 |
| 0.389 | 1.476 | 1.118 | 1.949 | 1.476 (1.118-1.949) | 0.006 | CRIP1 |
| 0.377 | 1.458 | 1.11 | 1.915 | 1.458 (1.110-1.915) | 0.007 | MMP25 |
| 0.371 | 1.449 | 1.106 | 1.899 | 1.449 (1.106-1.899) | 0.007 | CD209 |
| 0.376 | 1.456 | 1.111 | 1.91 | 1.456 (1.111-1.910) | 0.007 | CXCR4 |
| 0.374 | 1.453 | 1.107 | 1.906 | 1.453 (1.107-1.906) | 0.007 | DPEP2 |
| 0.367 | 1.444 | 1.102 | 1.892 | 1.444 (1.102-1.892) | 0.008 | ARHGDIB |
| 0.369 | 1.446 | 1.099 | 1.901 | 1.446 (1.099-1.901) | 0.008 | MYO1G |
| 0.365 | 1.441 | 1.099 | 1.89 | 1.441 (1.099-1.890) | 0.008 | SOWAHD |
| 0.366 | 1.442 | 1.1 | 1.889 | 1.442 (1.100-1.889) | 0.008 | FAM49A |
| 0.365 | 1.441 | 1.098 | 1.889 | 1.441 (1.098-1.889) | 0.008 | CCDC69 |
| 0.373 | 1.452 | 1.104 | 1.909 | 1.452 (1.104-1.909) | 0.008 | RP11-325F22.2 |
| 0.365 | 1.441 | 1.097 | 1.892 | 1.441 (1.097-1.892) | 0.009 | DEF6 |
| 0.364 | 1.438 | 1.096 | 1.888 | 1.438 (1.096-1.888) | 0.009 | DERL3 |
| 0.362 | 1.437 | 1.096 | 1.883 | 1.437 (1.096-1.883) | 0.009 | BTG2 |
| 0.362 | 1.436 | 1.096 | 1.881 | 1.436 (1.096-1.881) | 0.009 | SSTR2 |
| 0.366 | 1.442 | 1.096 | 1.898 | 1.442 (1.096-1.898) | 0.009 | RP5-1028K7.2 |
| 0.358 | 1.43 | 1.091 | 1.874 | 1.430 (1.091-1.874) | 0.01 | AMPD3 |
| 0.357 | 1.428 | 1.091 | 1.871 | 1.428 (1.091-1.871) | 0.01 | RBP5 |
| 0.35 | 1.419 | 1.083 | 1.859 | 1.419 (1.083-1.859) | 0.011 | STK17B |
| 0.355 | 1.426 | 1.084 | 1.877 | 1.426 (1.084-1.877) | 0.011 | NFKBIA |
| 0.356 | 1.428 | 1.086 | 1.877 | 1.428 (1.086-1.877) | 0.011 | FAM177B |
| 0.351 | 1.421 | 1.083 | 1.865 | 1.421 (1.083-1.865) | 0.011 | IGKV4-1 |
| 0.352 | 1.421 | 1.081 | 1.87 | 1.421 (1.081-1.870) | 0.012 | ANXA2R |
| 0.348 | 1.416 | 1.078 | 1.859 | 1.416 (1.078-1.859) | 0.012 | FMNL1 |
| 0.352 | 1.422 | 1.082 | 1.868 | 1.422 (1.082-1.868) | 0.012 | IL3RA |
| 0.348 | 1.416 | 1.078 | 1.859 | 1.416 (1.078-1.859) | 0.012 | RP11-405M12.4 |
| 0.344 | 1.41 | 1.076 | 1.849 | 1.410 (1.076-1.849) | 0.013 | SIDT1 |
| 0.345 | 1.412 | 1.077 | 1.851 | 1.412 (1.077-1.851) | 0.013 | SMAP2 |
| 0.34 | 1.405 | 1.073 | 1.84 | 1.405 (1.073-1.840) | 0.013 | SIRPB1 |
| 0.345 | 1.412 | 1.076 | 1.851 | 1.412 (1.076-1.851) | 0.013 | GATA3 |
| 0.344 | 1.411 | 1.075 | 1.851 | 1.411 (1.075-1.851) | 0.013 | P2RX1 |
| 0.342 | 1.408 | 1.075 | 1.846 | 1.408 (1.075-1.846) | 0.013 | ELMO1 |
| 0.343 | 1.409 | 1.075 | 1.848 | 1.409 (1.075-1.848) | 0.013 | GPR132 |
| 0.343 | 1.409 | 1.073 | 1.849 | 1.409 (1.073-1.849) | 0.014 | SH2D2A |
| 0.346 | 1.413 | 1.074 | 1.86 | 1.413 (1.074-1.860) | 0.014 | RASGRP2 |
| 0.338 | 1.402 | 1.07 | 1.837 | 1.402 (1.070-1.837) | 0.014 | ABCC3 |
| 0.342 | 1.407 | 1.073 | 1.845 | 1.407 (1.073-1.845) | 0.014 | PTPRN2 |
| 0.339 | 1.404 | 1.068 | 1.845 | 1.404 (1.068-1.845) | 0.015 | PIK3IP1 |
| 0.339 | 1.404 | 1.069 | 1.843 | 1.404 (1.069-1.843) | 0.015 | GZMM |
| 0.333 | 1.395 | 1.063 | 1.83 | 1.395 (1.063-1.830) | 0.016 | ATP2A3 |
| 0.332 | 1.394 | 1.063 | 1.828 | 1.394 (1.063-1.828) | 0.016 | CFP |
| 0.339 | 1.404 | 1.065 | 1.851 | 1.404 (1.065-1.851) | 0.016 | ANO9 |
| 0.337 | 1.401 | 1.064 | 1.844 | 1.401 (1.064-1.844) | 0.016 | CCNI2 |
| 0.329 | 1.39 | 1.061 | 1.821 | 1.390 (1.061-1.821) | 0.017 | CASP10 |
| 0.337 | 1.401 | 1.063 | 1.845 | 1.401 (1.063-1.845) | 0.017 | LGALS2 |
| 0.334 | 1.396 | 1.06 | 1.838 | 1.396 (1.060-1.838) | 0.017 | CTD-2020K17.1 |
| 0.329 | 1.389 | 1.058 | 1.824 | 1.389 (1.058-1.824) | 0.018 | SNAI3 |
| 0.326 | 1.385 | 1.055 | 1.818 | 1.385 (1.055-1.818) | 0.019 | TNFSF14 |
| 0.326 | 1.385 | 1.056 | 1.818 | 1.385 (1.056-1.818) | 0.019 | CCR7 |
| 0.32 | 1.377 | 1.051 | 1.805 | 1.377 (1.051-1.805) | 0.02 | IRF5 |
| 0.321 | 1.378 | 1.052 | 1.805 | 1.378 (1.052-1.805) | 0.02 | SYK |
| 0.323 | 1.382 | 1.048 | 1.822 | 1.382 (1.048-1.822) | 0.022 | GHRL |
| 0.316 | 1.372 | 1.046 | 1.799 | 1.372 (1.046-1.799) | 0.022 | DENND2D |
| 0.315 | 1.37 | 1.044 | 1.8 | 1.370 (1.044-1.800) | 0.023 | TNFAIP2 |
| 0.314 | 1.369 | 1.044 | 1.795 | 1.369 (1.044-1.795) | 0.023 | LILRA4 |
| 0.315 | 1.371 | 1.044 | 1.8 | 1.371 (1.044-1.800) | 0.023 | SPIB |
| 0.309 | 1.362 | 1.039 | 1.785 | 1.362 (1.039-1.785) | 0.025 | RASGEF1B |
| 0.311 | 1.365 | 1.041 | 1.791 | 1.365 (1.041-1.791) | 0.025 | RP11-93B14.9 |
| 0.309 | 1.362 | 1.038 | 1.786 | 1.362 (1.038-1.786) | 0.026 | MAP4K1 |
| 0.307 | 1.36 | 1.037 | 1.783 | 1.360 (1.037-1.783) | 0.026 | CAMK1D |
| 0.307 | 1.359 | 1.038 | 1.78 | 1.359 (1.038-1.780) | 0.026 | IGHV1-46 |
| 0.305 | 1.356 | 1.035 | 1.778 | 1.356 (1.035-1.778) | 0.027 | PLA2G7 |
| 0.3 | 1.35 | 1.031 | 1.769 | 1.350 (1.031-1.769) | 0.029 | TCL1A |
| 0.308 | 1.36 | 1.031 | 1.794 | 1.360 (1.031-1.794) | 0.029 | IGFLR1 |
| 0.302 | 1.352 | 1.032 | 1.772 | 1.352 (1.032-1.772) | 0.029 | GCSAM |
| 0.299 | 1.349 | 1.028 | 1.77 | 1.349 (1.028-1.770) | 0.031 | HVCN1 |
| 0.299 | 1.348 | 1.028 | 1.768 | 1.348 (1.028-1.768) | 0.031 | GRB2 |
| 0.296 | 1.344 | 1.024 | 1.766 | 1.344 (1.024-1.766) | 0.033 | RHOF |
| 0.298 | 1.347 | 1.024 | 1.773 | 1.347 (1.024-1.773) | 0.033 | RP4-647J21.1 |
| 0.293 | 1.34 | 1.022 | 1.757 | 1.340 (1.022-1.757) | 0.034 | PRKCB |
| 0.29 | 1.337 | 1.019 | 1.753 | 1.337 (1.019-1.753) | 0.036 | ITGB7 |
| 0.295 | 1.344 | 1.02 | 1.77 | 1.344 (1.020-1.770) | 0.036 | SPNS3 |
| 0.285 | 1.33 | 1.014 | 1.743 | 1.330 (1.014-1.743) | 0.039 | IGHV5-78 |
| 0.28 | 1.323 | 1.009 | 1.734 | 1.323 (1.009-1.734) | 0.043 | DHRS9 |
| 0.274 | 1.315 | 1.002 | 1.726 | 1.315 (1.002-1.726) | 0.048 | CLU |
| 0.275 | 1.317 | 1.003 | 1.729 | 1.317 (1.003-1.729) | 0.048 | CBFA2T3 |
| 0.272 | 1.312 | 1.001 | 1.72 | 1.312 (1.001-1.720) | 0.049 | ICAM2 |
| 0.275 | 1.317 | 1 | 1.735 | 1.317 (1.000-1.735) | 0.05 | UNC13D |
| 0.268 | 1.308 | 0.997 | 1.715 | 1.308 (0.997-1.715) | 0.053 | VOPP1 |
| 0.266 | 1.304 | 0.994 | 1.712 | 1.304 (0.994-1.712) | 0.056 | AKNA |
| 0.261 | 1.299 | 0.991 | 1.701 | 1.299 (0.991-1.701) | 0.058 | CHST15 |
| 0.252 | 1.286 | 0.982 | 1.685 | 1.286 (0.982-1.685) | 0.068 | FUCA1 |
| 0.249 | 1.283 | 0.979 | 1.683 | 1.283 (0.979-1.683) | 0.071 | PLCG2 |
| 0.24 | 1.272 | 0.971 | 1.666 | 1.272 (0.971-1.666) | 0.081 | TTC9 |
| 0.24 | 1.272 | 0.971 | 1.665 | 1.272 (0.971-1.665) | 0.081 | S1PR1 |
| 0.239 | 1.27 | 0.967 | 1.667 | 1.270 (0.967-1.667) | 0.085 | HMHA1 |
| 0.24 | 1.271 | 0.967 | 1.671 | 1.271 (0.967-1.671) | 0.086 | BATF |
| 0.237 | 1.267 | 0.966 | 1.661 | 1.267 (0.966-1.661) | 0.087 | ARHGAP27 |
| 0.234 | 1.264 | 0.963 | 1.659 | 1.264 (0.963-1.659) | 0.092 | SLC22A3 |
| 0.228 | 1.256 | 0.959 | 1.645 | 1.256 (0.959-1.645) | 0.098 | NECAP2 |
| 0.227 | 1.254 | 0.958 | 1.643 | 1.254 (0.958-1.643) | 0.1 | CXCL12 |
| 0.217 | 1.242 | 0.948 | 1.629 | 1.242 (0.948-1.629) | 0.116 | PRKCH |
| 0.217 | 1.242 | 0.948 | 1.629 | 1.242 (0.948-1.629) | 0.116 | TTC7A |
| 0.219 | 1.244 | 0.947 | 1.635 | 1.244 (0.947-1.635) | 0.117 | NUAK2 |
| 0.216 | 1.241 | 0.946 | 1.629 | 1.241 (0.946-1.629) | 0.119 | DOK3 |
| 0.213 | 1.238 | 0.944 | 1.623 | 1.238 (0.944-1.623) | 0.123 | PIM1 |
| 0.209 | 1.232 | 0.939 | 1.618 | 1.232 (0.939-1.618) | 0.133 | LYL1 |
| 0.201 | 1.223 | 0.934 | 1.601 | 1.223 (0.934-1.601) | 0.144 | ABI3BP |
| 0.2 | 1.222 | 0.932 | 1.601 | 1.222 (0.932-1.601) | 0.146 | RILPL2 |
| 0.201 | 1.223 | 0.931 | 1.607 | 1.223 (0.931-1.607) | 0.149 | CCL19 |
| 0.197 | 1.218 | 0.927 | 1.6 | 1.218 (0.927-1.600) | 0.156 | FCHO1 |
| 0.192 | 1.211 | 0.921 | 1.593 | 1.211 (0.921-1.593) | 0.171 | SYTL1 |
| 0.187 | 1.206 | 0.92 | 1.58 | 1.206 (0.920-1.580) | 0.175 | 6-Sep |
| 0.184 | 1.202 | 0.918 | 1.576 | 1.202 (0.918-1.576) | 0.182 | CCDC88C |
| 0.184 | 1.203 | 0.913 | 1.583 | 1.203 (0.913-1.583) | 0.189 | EVL |
| 0.176 | 1.192 | 0.91 | 1.561 | 1.192 (0.910-1.561) | 0.202 | CD79B |
| 0.161 | 1.175 | 0.895 | 1.541 | 1.175 (0.895-1.541) | 0.245 | RFTN1 |
| 0.16 | 1.174 | 0.895 | 1.541 | 1.174 (0.895-1.541) | 0.247 | CYTH1 |
| 0.156 | 1.169 | 0.893 | 1.531 | 1.169 (0.893-1.531) | 0.256 | RP11-389C8.2 |
| 0.149 | 1.161 | 0.886 | 1.521 | 1.161 (0.886-1.521) | 0.28 | SLC46A3 |
| 0.143 | 1.154 | 0.879 | 1.514 | 1.154 (0.879-1.514) | 0.301 | SH2D3C |
| 0.138 | 1.148 | 0.875 | 1.506 | 1.148 (0.875-1.506) | 0.318 | IL4R |
| 0.132 | 1.141 | 0.87 | 1.496 | 1.141 (0.870-1.496) | 0.342 | DNASE1L3 |
| 0.129 | 1.137 | 0.869 | 1.489 | 1.137 (0.869-1.489) | 0.349 | BASP1 |
| 0.0721 | 1.075 | 0.821 | 1.407 | 1.075 (0.821-1.407) | 0.6 | PECAM1 |
| 0.0686 | 1.071 | 0.815 | 1.407 | 1.071 (0.815-1.407) | 0.622 | PPP1R16B |
| 0.0435 | 1.044 | 0.797 | 1.368 | 1.044 (0.797-1.368) | 0.752 | GRAP |
| 0.019 | 1.019 | 0.778 | 1.335 | 1.019 (0.778-1.335) | 0.89 | ANKRD33B |
| -0.017 | 0.983 | 0.751 | 1.288 | 0.983 (0.751-1.288) | 0.902 | CCL21 |
| 0.0126 | 1.013 | 0.772 | 1.328 | 1.013 (0.772-1.328) | 0.927 | GMIP |

| **Supplemental Table S4. Association between m6a cluster and m6a signature subtype** | | | | |
| --- | --- | --- | --- | --- |
|  | **m6a-C1** | **m6a-C2** | **m6a-C3** | **adjusted χ2 test** |
| **m6Sig-SI** | 132 | 10 | 3 | p < 0.0001 |
| **m6Sig-SII** | 0 | 33 | 87 |  |
| **m6Sig-SIII** | 53 | 133 | 2 |  |

## Supplementary Codes

## # Nonnegative matrix factorization (NMF)

**library(NMF)**

**res <- nmf(gene_expression_matrix, 2:6,**

**nrun = 200, method = 'brunet')**

**plot(res)**

**consensusmap(res,labCol=NA, labRow=NA)**

**fit <- nmf(gene_expression_matrix, 3, nrun =**

**200, method = "brunet")**

**cluster.result <- predict(fit)**

**# Gene set variation analysis (GSVA)**

**library(GSVA)**

**library(ggplot2)**

**gsva_es <- gsva(gene_expression_data, list=pathway.list,**

**method='GSVA',kcdf='Gaussian',abs.ranking=F)**

**# Waterfall and gene mutation plot**

**library(maftools)**

**library(BSgenome.Hsapiens.UCSC.hg19)**

**maf_data <- read.maf(maf="./data.maf",clinicalData = cli)**

**oncoplot(maf = maf_data,genes= genes_to_plot, draw_titv = TRUE,**

**borderCol=NULL,clinicalFeatures = clin_var)**

**# Visualization of co-occurrence and exclusion**

**somaticInteractions(maf=maf_data, genes =**

**genes_to_compare, pvalue=c(0.05, 0.01))**

**# Identification of differential expression genes(DEG)**

**library(limma)**

**design <- model.matrix(~0+factor(cli$subtype_group))**

**colnames(design) <- c("c1","c2","c3")**

**fit <- lmFit(gene_expression_data, design)**

**contrast.matrix <- makeContrasts(c1-c2,c1-c3,c2-c3,levels = design)**

**fit1 <- contrasts.fit(fit,contrast.matrix)**

**fit1 <- eBayes(fit1)**

**DEG <- decideTests(fit1)**

**# Survival and Multivariate regression model**

**library(forestmodel)**

**library(survminer)**

**survfit <- survfit(Surv(survival_time, survival_status)~subtype_group, cli)**

**ggsurvplot(survfit,pval = TRUE,risk.table = TRUE,**

**palette = color_code, ggtheme = theme_bw())**

**forest_model(coxph(Surv(survival_time, survival_status) ~**

**variables, related_data), factor_separate_line = T)**

**# Construct the score**

**library(caret)**

**data1 <- gene_expression_data[rownames(gene_expression_data) %in%**

**prognosis_gene,]**

**data1 <- as.data.frame(t(data1))**

**control <- rfeControl(functions=rfFuncs, method="cv", number=10)**

**rfe_res <- rfe(data1,as.factor(cli$subtype_group), rfeControl=control)**

**data2 <- gene_expression_data[rownames(gene_expression_data) %in%**

**rfe_res$optVariables, ]**

**data2 <- scale(t(data2))**

**score <- princomp(data2,cor = T)**

**score <- apply(score$scores[,1:2],1,sum)**

**# Correlation plot**

**library(corrplot)**

**cor_mat <- cor(gene_expression_matrix,immune_signautre,**

**method = "pearson")**

**cor_p<-corr.test(gene_expression_matrix,immune_signautre,**

**method = "pearson")$p**

**corrplot(cor_mat, p.mat = cor_p, method = "color", type = "full",col=heat_color,**

**sig.level = c(.001, .01, .05), pch.cex = .7,tl.col = "black",tl.srt = 45,**

**insig = "label_sig", pch.col ="black")**
